# In-school eyecare in special education settings has measurable benefits for children’s vision and behaviour

**DOI:** 10.1371/journal.pone.0220480

**Published:** 2019-08-01

**Authors:** S. A. Black, E. L. McConnell, L. McKerr, J. F. McClelland, J. A. Little, K. Dillenburger, A. J. Jackson, P. M. Anketell, K. J. Saunders

**Affiliations:** 1 School of Biomedical Sciences, Optometry and Vision Sciences, Ulster University, Coleraine, Northern Ireland; 2 School of Social Sciences, Education and Social Work, Queen’s University, Belfast, Northern Ireland; 3 Health and Social Care Trust, Belfast, Northern Ireland; Universtiyt of Oviedo (Spain), SPAIN

## Abstract

**Objectives:**

To determine whether implementation of comprehensive in-school eyecare results in measurable benefits for children and young people in terms of visual status, classroom behaviours and how well their visual needs are met.

**Design:**

School-based observational study.

**Participants & Methods:**

200 pupils [mean age 10 years 9 months, 70% male, majority moderate (40%) or severe (35%) learning difficulty] of a special education school in the UK.

A sector-agreed in-school eyecare framework including full eye examination and cycloplegic refraction, dispensing of spectacles (as appropriate) and written reporting of outcomes to parents/teachers was applied. Classroom behaviours were observed and recorded prior to, and after, the in-school eyecare. Surveys were employed to obtain visual histories from parents/teachers. School records and statutory documents were reviewed for diagnostic and learning disability classifications. Visual function and ocular health were profiled at baseline and significant visual deficits identified. Where such deficits were previously unrecognised, untreated or not compensated for (e.g. correction of refractive error, enlargement of educational material) they were recorded as ‘unmet visual need’. At follow-up, 2–5 months after initial (baseline) measures, eye examinations, parent/teacher surveys and behaviour observations were repeated. Follow-up measures were used to determine if measurable improvements were evident in visual function, ocular health, the level of unmet need and classroom behaviour following implementation of in-school eyecare.

**Results:**

199 participants completed baseline and follow-up measures. 122 (61%) participants presented with at least one significant visual or ocular health deficit and 90 (45%) participants had at least one unmet visual need. Younger pupils and those with no previous history of eyecare were more likely to demonstrate unmet visual needs at baseline (OR 1.12 95% CI 1.03 to 1.21) p = 0.012; (OR 4.44 95% CI 1.38 to 14.29 p = 0.007 respectively). On follow-up, the number of pupils with unmet visual needs dropped significantly to 36 (18%) (McNemar’s test p<0.001). Visual and behavioural metrics of participants without significant visual deficits or whose visual needs were adequately addressed at baseline remained relatively unchanged between baseline and follow-up (Wilcoxon signed rank p>0.05). Where significant refractive deficits were corrected at follow-up, near visual acuity improved significantly (Wilcoxon signed rank p = 0.013), however, poor spectacle compliance was a persistent cause of unmet visual need. Off-task behaviour reduced significantly after actions to address unmet visual needs were communicated to parents and teachers (Wilcoxon signed rank p = 0.035).

**Conclusions:**

The present study demonstrates for the first time measurable visual and behaviour benefits to children in special education settings when they receive comprehensive in-school eye examinations, on-site spectacle dispensing and jargon-free reporting of outcomes to teachers and parents.

## Introduction

It is evident from the literature that children with developmental disability are at a higher risk of visual problems compared to typically developing children [1–7] and UK charity SeeAbility report that children with learning disability are 28 times more likely to have a serious sight problem compared to their typically developing peers [8]. A variety of reports identify that this group of vulnerable children may have difficulty in accessing eyecare services and that where services are accessed, vital information regarding their visual status is not transferred in a meaningful way to education providers [1,9–11].

In-school vision checks (limited to measurement of distance vision in either eye) are recommended by Public Health England in the UK as a screening tool suitable for typically developing children in mainstream education settings. However, due to the increased risk of visual impairment, vision screening is not appropriate for children in special education settings [12,13] and instead comprehensive eye examinations are recommended [14].

However, access to eyecare can be challenging for children with developmental disabilities and their families. As a means to promote equitable access to regular eyecare, key eyecare stakeholders and charities in the UK have collaboratively designed a framework for in-school eyecare for special educational settings [15] and the Clinical Council for Eye Health Commissioning has recently given its endorsement for a comprehensive and targeted programme of eyecare for children and young people in special schools in England. This in-school eyecare framework aims to ensure children with special educational needs have access to eyecare, including comprehensive eye examinations and dispensing of spectacles, in a familiar setting; and that parents, teachers and other stakeholders receive meaningful information to support children’s visual needs at home and school.

The present study aimed to determine, for the first time, whether implementation of a comprehensive in-school eyecare framework results in measurable benefits for children in terms of vision and classroom behaviours.

## Participants and methods

Ethical approval for the study was gained through Ulster University Research Ethics Committee (REC/15/0125) and the study adhered to the Tenets of the Declaration of Helsinki.

### Recruitment

**Fig 1:**
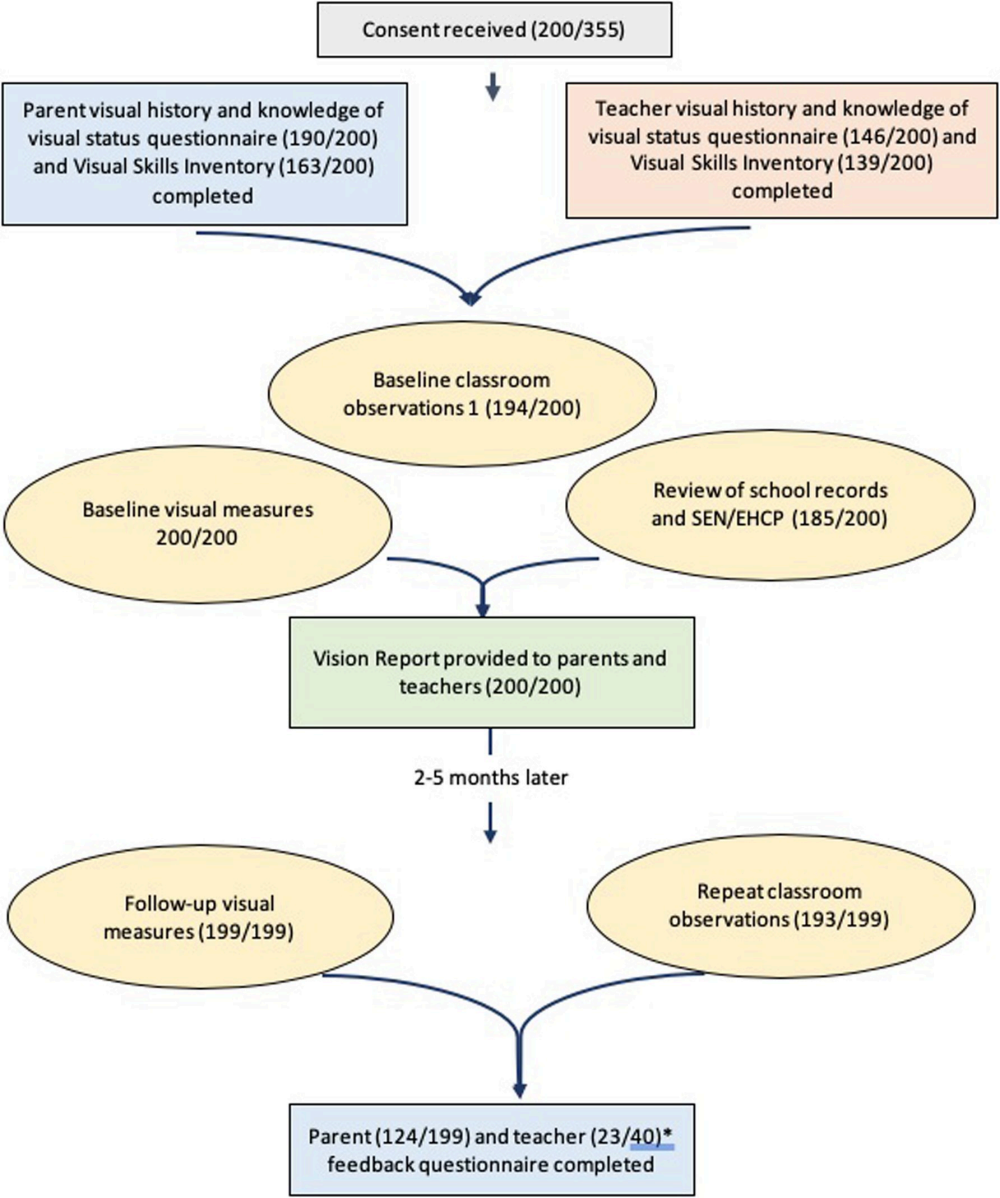
Summary of the research process and number of participants at each stage. *Many participants were in the same class and, therefore, teachers completed feedback questionnaires on more than one participant.

All parents of children and young people attending Castle Tower School were contacted to invite them and their child or young person to participate in the study. Castle Tower school is the largest special education school in Northern Ireland (a region of the United Kingdom), with 335 pupils ranging from mild/moderate to profound learning disability. Permission was also sought for the research team to contact teaching staff and to access the child or young person’s school medical and educational records. Contact was made through the school, with permission of the Principal. Data collection took place September 2016 through June 2018, excluding school holidays. Fig 1 outlines the research process.

### Baseline measures—Parents and teaching staff

Parents and teaching staff were questioned regarding several aspects of each participant’s medical and ocular history and visual status. A member of the research team worked with parents to complete surveys and inventories either over the telephone, or in person, depending on preference. Teaching staff undertook these tasks independently, however, they were able to ask the research team for assistance, as required.

#### Visual history and knowledge of visual status

A written clinical history questionnaire was given to parents in order to establish:

- The participant’s ophthalmic and brief medical history, including where and when they had their last eye appointments, history of glasses wear and any parental concerns about vision.
- Parental awareness or concerns about participant’s visual function or ocular health
- Modifications made at home to account for any visual deficit, if known.

Teaching staff were issued with a written questionnaire to complete for each participant for whom consent had been gained. Information was obtained regarding:

- The teaching staff’s awareness of any visual problems or limitations pertaining to the participant.
- Any modifications or adjustments currently in place to reduce the impact of each participant’s visual deficits or difficulties in the classroom

#### Evidence of visual processing difficulties

To explore visual behaviours which may indicate visual processing difficulties, parents and teaching staff were asked to complete the Visual Skills Inventory (VSI) developed by Dutton and colleagues [16] for each participant.

Participants’ school medical records and Statement of Educational Need (Education Health and Care Plan) were reviewed to determine the primary diagnosis and level of learning disability at the time of participation.

### Baseline measures–Participants

#### Classroom engagement

Before any visual measures were undertaken, classroom observations were conducted by an experienced observer (LMcK), trained and monitored by a behaviour analyst (KD). Observations were scheduled to optimise the circumstances in which identified target behaviours could occur [17] i.e. periods of structured engagement in the learning environment. Note was taken of each participant’s position in the classroom with respect to general learning resources such as TV screens and whiteboards. Each participant was observed over a period of ten minutes, using a time-sampling methodology. The number of specific behaviours occurring during one-minute intervals were noted [18]. The following target behaviours were chosen as appropriate behaviours with which to profile classroom engagement across the wide range of participant learning abilities and teaching methodologies employed across the school. Behaviours were documented using a modified check-sheet employed in the ‘Good Inclusion Game’ [19]:

- Initiates engagement with peer/teacher*
- Off task (defined as an event where a pupil disengages from the set task)
- Teacher’s positive comment in response to pupil’s behaviour
- Not visually engaged with task
- Repetitive behaviours
- Wearing spectacles during task

*’Teacher’ refers to all staff members in classroom, including classroom assistants.

The check-sheet was amended, with permission from the authors’ of the Good Inclusion Game, to reflect the needs and skills of participants who were pre-verbal and/or had limited communication skills.

#### Visual and ocular health assessment

A full eye examination was undertaken on all participants in a suitable, well illuminated room of minimum 3 metres in length and which could be blacked out. The following measures which were used to identify significant baseline visual deficits (see Table 1 for further detail): *Habitual visual acuity* at distance and near using tests appropriate for the participant’s age and ability.

- Contrast sensitivity
- Accommodative function
- *Ocular alignment* using cover test at distance and near
- *Ocular movement assessment* including fixation quality and extent, quality and control of eye movements
- Binocular visual field assessment
- Refractive error (cycloplegic retinoscopy)
- Dilated internal and external ocular health assessment

Where formal tests of visual acuity failed to elicit a response, the Bradford Visual Function Box [20] was used to gain an indication of visual function.

**Table 1:** Tests attempted to ascertain visual status and success rates achieved at baseline.

*Test*		*Method*	*Success rate % (n)*
Vision	Distance	Formal measurement of vision successfully achieved*	**98.5% n = 197**
		Sonsken crowded logMAR letters at 3m	60.9% *(120/197)*
		Sonsken logMAR single letters at 3m	0.5% (*1/197*)
		LEA crowded logMAR symbols at 3m	13.7% *(27/197)*
		LEA logMAR single symbols at 3m	1.5% *(3/197)*
		Cardiff acuity test	23.9% *(47/197*)
	Near	**Formal measurement of vision successfully achieved**	**70.5% n = 141**
		Sonsken crowded logMAR letters at 40cm	82.3% (116/141)
		LEA crowded logMAR symbols at 40cm	17.7% (25/141)
Ocular alignment	Distance	Prism cover test (3m)	99% (n = 198)
	Near	Prism cover test (40cm)	100% (n = 200)
Ocular movements		Pursuit and saccadic eye movement quality to penlight at 40cm	88.5% (n = 177)
		Ocular movements in eight directions of gaze	84% (n = 168)
		Near point of convergence to target, until break noted or diplopia reported	88.5% (n = 177)
Accommodative function		Dynamic retinoscopy (Ulster-Cardiff accommodation CUBE with target at 25cm/4D)	97% (n = 194)
Contrast sensitivity		Cardiff Contrast Test	91.5% (n = 183)
Visual Field		Binocular gross confrontation to a 15cm white ball	93.5% (n = 187)
Refractive error		Cycloplegic retinoscopy (1% cyclopentolate HCl)	91.9% (181/197)
		Non-cycloplegic distance static retinoscopy	8.1% (16/197)
		**All methods**	**98.5% (n = 197)**
Ocular health		Dilated direct/indirect ophthalmoscopy	91.8% (181/197)
		Un-dilated direct/indirect ophthalmoscopy	8.1% (16/197)
		**All methods**	**99.5% (n = 199)**
Visual Processing		LEA crowded logMAR symbols at 3m	73.5% (n = 147)
		LEA logMAR single symbols at 3m	
		Profiled using the Visual Skills Inventory (VSI) completed by parents and teachers	75.5% (n = 151)
		**All methods**	**91.5% (n = 183)**

* The Bradford Visual Function Box was used to gain an indication of visual function when a participant was not able to engage with a formal measure of vision.

In addition to gathering information from parents about visual processing deficits through the VSI, measurement of crowded and single binocular acuity using LEA symbols at 3 metres was attempted during the in-school eye examination. The difference between crowded and single measures were compared to further explore evidence of difficulties with processing ‘crowded’ visual information, a common feature of visual processing difficulties amongst children with developmental disability [21–23].

Eye examinations took place during the school day and parents were invited to attend. If necessary, eye examinations could be completed over more than one in-school testing session.

#### In-school spectacle dispensing

A range of suitable spectacle frames were available to fit, glaze and dispense up-dated or new spectacle corrections where required. The costs of spectacles and lenses were covered by the research funding. Parents were involved in the choice of frame. Alternatively, parents could take spectacle prescriptions to their community optometrist, if preferred. Where participants had a current spectacle correction, updated prescriptions were shared with the provider.

#### Reporting

Following baseline measures, parents and teachers were supplied with a semi-standardised written ‘Vision Report’ describing the participant’s visual status in lay terms [24,25]. Reports were also shared with other health and care providers, as appropriate. The Vision Report identified visual strengths and weaknesses and highlighted actions needed to optimise visual potential and address unmet visual needs identified through the parent/teacher questionnaires, VSI and eye examination. For example, the Vision Report contained advice and actions regarding:

- Requirements for new or updated spectacle wear and recommended wearing schedule
- Advice on appropriate classroom and home modifications e.g. enlarged print, high contrast educational materials, seating position, reducing clutter etc.
- Strategies to manage signs of visual processing difficulties identified through the VSI
- Requirement for referral to specialist services, e.g. ophthalmological attention and details on the referral made.

Eye examinations, dispensing and fitting of spectacles and report-writing was undertaken by two optometrists (ELM, SAB), experienced in paediatric assessment.

### Follow-up measures- Participants

Two to five months after the Vision Report was provided, follow-up measures were instigated. This time period allowed sufficient time for the report to be received, suggested actions/advice to be implemented, whilst limiting the impact of the participant’s natural development on visual and behavioural metrics. The following measures were repeated;

- Classroom Engagement: observations repeated by same observer, masked to outcomes of baseline eye examination and Vision Report.
- Eye examination: repeat measures of visual status, including distance and near visual acuity, refractive error and accommodative response.

### Follow-up measures: Parents and teaching staff

Feedback questionnaires were issued to parents and teachers after the follow-up assessment. To ensure maximal return rates of the questionnaires, reminders were issued to parents and teachers in person, over the phone, via text message or via email. These questionnaires were used to determine whether appreciation of the participants’ visual status had altered and whether actions recommended in the Vision Report had been implemented i.e. whether spectacles were worn, learning material adapted, environmental modifications made.

### Statistical approach

All statistical analyses were performed using (IBM SPSS Statistics for Macintosh, Version 25.0. Armonk, NY:IBM Corp 2017). Categorical variables were summarised by frequencies and percentages and the Shapiro-Wilk test was applied to test whether data were distributed normally. Count data obtained through behavioural observations were summarised by median and IQR (inter-quartile range). McNemar’s test was used to evaluate differences in the number of participants demonstrating dichotomous traits at baseline compared with follow-up and multiple logistic regression analyses were used to investigate the association between dichotomous traits at baseline and independent variables such as age, level of learning disability and previous history of eyecare. Pearson’s correlation was used to investigate relationships between normally distributed related continuous variables. Changes in paired metrics (non-parametric distributions) were evaluated using Wilcoxon signed ranked test. One-tailed tests were used when testing the hypothesis that visual status or behaviour had improved between baseline and follow-up measures.

Das et al (2010) report that 24% of their sample of 228 children in a special education setting presented with uncorrected, or sub-optimally corrected, refractive error (as determined by cycloplegic retinoscopy) and requiring a new/updated prescription. To determine a reduction of 50% or more in this metric, with a statistical power of 95%, required a sample size of 106. This sample size was inflated, to allow for drop-outs at follow-up.

## Results

### Participant profile

Consent was obtained for 200 of the 335 pupils enrolled in the school; representing a 59.7% consent rate. Participants were aged from 3 years 7 months to 19 years 9 months (mean age 10 years 9 months), 70% were male.

According to school medical and statutory records, participants were diagnosed with a range of medical conditions and syndromes including Autistic spectrum disorders (33%), Down syndrome (9.7%) and cerebral palsy (2.7%). The majority were reported by parents to be born at (or near) term (78.2%), with 15.3% born prematurely (before 37 weeks). According to records, the level of learning difficulty ranged from Profound (1.2%; n = 2) to Mild/Moderate (0.5%; n = 1). The majority of participants had either Severe (35%; n = 70) or Moderate (40%; n = 80) learning difficulties. The sample was representative of the pupil profile of the school in terms of gender, level of learning disability and age (Chi-square p = 0.446, p = 0.722 LD and Mann-Whitney U for age p = 0.053 respectively).

One participant withdrew from the study after the baseline measures. No reason was given for withdrawal.

#### Visual history

Visual histories were available for 190 (95%) of participants through parent survey. According to parent report, 11.1% (n = 21) of participants had no previous history of eyecare and eyecare history was unknown for 1.1% (n = 2) participants. Of those participants for whom a positive history of eyecare was reported (n = 167), half had received their last eye examination in a hospital clinic (50.2%, n = 84), 37.7% (n = 63) in community optometry practices and four were reported as having received in-school eyecare. The remainder were unsure of the setting of the last eye examination (10.2%, n = 17). The last eye examination had occurred within the previous two years for 66.5% of participants with a history of eyecare. Three participants were registered as severely sight impaired.

#### Success rates for baseline eye examination

Seventy-one percent of participants (n = 142) completed the baseline eye examination in one sitting, 29% (n = 58) required repeat sessions (52 two sessions, 5 three sessions, 1 four sessions). At follow-up, 192 (96.5%) participants completed the eye examination in one session and the remaining seven needed one further session to complete. Table 1 details the tests used to assess visual status and the success rates achieved.

### Baseline/Presenting visual profile of participants

#### Presenting visual impairment

A formal measure of distance visual acuity was achieved for all but three participants. The remaining participants were assessed using the Bradford Visual Function Box. These participants’ data were not included in the analysis of presenting visual impairment. Distance visual impairment was identified using the criteria of Cumberland et al (2016) and the World Health Organisation (ICD-11, 2018) i.e. a presenting visual acuity of poorer than 0.3logMAR (either binocular or in their best eye). Near visual impairment was identified using the World Health Organisation (ICD-11, 2018) criteria, i.e. a presenting near acuity of 0.4logMAR or poorer. Twelve (6.1%; 12/197) participants presented with a distance visual impairment, seven (5.0%; 7/141) had a presenting near visual impairment and two were impaired at both distance and near. In ten cases of impaired vision (distance or near), these visual impairments were unknown to the parents or teacher, according to visual history-taking. These participants’ visual impairments were not being considered in the classroom or at home, and no modifications had been made to educational or recreational materials to accommodate reduced vision. Three participants had acuity of 1.0logMAR or poorer, meeting the threshold for registration as severely sight-impaired; all were identified as being appropriately certified through parent report and in the statutory document detailing their educational support needs.

#### Refractive error and accommodation

A large range of ametropia (SER –14.00D to +9.00D) and astigmatism were found in this cohort, consistent with the literature for children with developmental disability. For the present analysis, criteria were required to enable consideration of refractive error as an unmet visual need. So, rather than consider refractive error using parameters appropriate to describe prevalence, a criterion was required where lack of refractive correction would mean a significant detriment to visual function. Accordingly, the conservative American Academy of Ophthalmology (AAO) guidelines for refractive correction of children over three years of age [26] was used to define **significant** uncorrected refractive error for the least ametropic eye (Table 2). These criteria were also used to prescribe and update spectacles at baseline. A more clinical approach was taken at follow-up to address lower levels of refractive error.

**Table 2:** Prevalence of significant refractive error in the present study.

**Significant Refractive Error** [26]	N (%)
Isometropia	
Myopia ≤-2.50	1 (0.5)
Hyperopia ≥+3.50 (No manifest deviation)	14 (7.0)
Hyperopia ≥+1.50 (with esotropia)	14 (7.0)
Astigmatism* ≥1.50DC	29 (14.5)
Anisometropia*	
Myopia ≤-2.50	5 (2.5)
Hyperopia ≥+1.50	15 (7.5)
Astigmatism ≥1.50DC	4 (2.0)
**Total with Significant Refractive Error (AAO criteria)**	**63 (32%)**

*Participants with these refractive errors may also be represented in spherical groups.

Sixty-three (32%) participants had significant refractive errors as defined by the AAO criteria; 20 of whom (31.7%) presented without correction. Of these, by parental report, two had no reported history of previous eyecare and eight, whilst having a previous history of eyecare, had never had spectacles.

Thirty-three (17%) participants presented with significantly reduced accommodative responses (hypo-accommodation) to a near target [27]; only four participants had evidence that this deficit was being appropriately managed at baseline (bifocal correction).

Twenty participants presented with both significant refractive error and hypo-accommodation; in 11 cases these deficits were uncorrected.

Participants who presented wearing appropriately powered spectacles to correct their refractive error and/or accommodative deficit or had no significant refractive error (as defined above) were regarded as having their refractive needs met at baseline (82.2%, n = 162/197). The remainder were regarded as having an ‘unmet need’ in relation to refractive error. Thirty-five participants needed a first time correction, updated spectacles, a bifocal correction or had a history of spectacle wear but presented unaided.

#### Contrast sensitivity

Twenty-four (13.1%) participants presented with reduced contrast sensitivity compared with published normative data [28]. In none of these cases were parents or teachers aware of the visual deficit and no compensations or modifications had been made in class or at home to reduce the impact of this deficit. Twenty-three (95.8%) participants with contrast deficits had a history of eyecare.

#### Visual field deficits

Gross confrontation revealed four (2%) participants with significant restrictions of their visual fields [29]; three presented with hemianopia and one participant demonstrated a general constriction of their visual field. Visual histories revealed that all had a history of eyecare but that parents and teachers were not aware of these visual field restrictions and no account was taken of them either at home or in the school environment to compensate for the deficit.

#### Eye movement control

Most participants had full ocular movements and normal pursuits, saccades and convergence. However, 20 (11.3%) participants demonstrated abnormal ocular movements, including seven with significantly reduced near point of convergence (NPC). All but one participant with abnormal eye movements had a previous history of eyecare but only seven parents appreciated the presence of abnormal ocular movements (35%).

#### Ocular health

Eighteen (9.0%) participants had one or more ocular anomaly (Table 3). While 17 had a history of previous eyecare, six were receiving no current treatment/management as parents were unaware of the condition.

**Table 3:** Anomalies of ocular health or structure noted during the internal and external ocular examination.

Condition	N (%)
Blocked tear ducts	3 (1.5)
Blepharitis	4 (2.0)
Lens anomalies (cataracts, subluxated lens, IOLs)	6 (3)
Ptosis	2 (1)
Tortuous blood vessels	1 (0.5)
Optic disc anomalies (pale disc, drusen)	2 (1)
Iris synechae	1 (0.5)
Retinal naevus	1 (0.5)

#### Ocular alignment

Thirty-nine (19.5%) participants had a manifest strabismus at distance or near or both and nine had nystagmus (Table 4).

**Table 4:** Prevalence and type of ocular alignment anomalies.

Type of Strabismus	Distance N (%)	Near N (%)
Esotropia	18 (9)	17 (8.5)
Exotropia	16 (8)	15 (7.5)
Hyper/hypotropia	2 (1)	2 (1)
Nystagmus	9 (4.5)	

#### Crowding deficits and evidence of visual processing difficulties

A total of 43 (23.5%) participants presented with a crowding deficit and/or evidence of visual processing difficulties. Thirty-one (16.9%) participants had evidence of crowding deficits identified through the Dutton Visual Skills Inventory (VSI) or where comparison of crowded binocular acuity was more than two lines poorer than the measure achieved using single optotypes [30] Evidence of difficulties with additional aspects of visual processing (beyond crowding issues), was reported through the VSI for 24 (15.9%) participants. Twelve participants demonstrated difficulties with both crowding and other aspects of visual processing.

Where evidence of visual processing deficits was revealed through the VSI and/or deficits in crowded versus single optotype acuity was found, there was no evidence of parents or teachers having previously been given strategies or information to support pupils in coping with these difficulties, except for one participant who had a diagnosis of cerebral visual impairment. Neither did parents report previous exploration of these difficulties by eye/health professionals prior to the in-school eye examination. Of the 43 participants identified as having a deficit, 41 (95.3%) had a previous history of eyecare.

#### Most common visual deficits

Refractive issues, including poor focus and significant ametropia, reduced contrast sensitivity and evidence of visual processing difficulties (including crowding) were the most common visual deficits identified through the in-school eye examination and application of the VSI. 122 (61%) participants presented with at least one significant visual deficit.

#### How well were visual needs met at baseline?

Where participants presented with a significant visual or ocular deficit as described above, data from the baseline eye examination and the visual histories were used to identify whether these deficits were known about and accounted for. Where this was not evidenced, the participant was defined as having ‘unmet visual need(s)’. Ninety participants (45.0%) presented with an unmet visual need (Table 5) at baseline; 31 had more than one unmet visual need and three presented with four unmet visual needs.

**Table 5:** Percentage (n) of participants presenting with significant visual deficits at baseline, the proportion of these visual deficits which were unrecognised and/or unaddressed and, as such, defined as an ‘unmet visual need’, at baseline and follow-up.

Visual deficit	% presenting with a significant visual deficit at baseline (n)	% with visual deficit and ‘unmet visual need’* (n)		McNemar’s chi-square test
Significant Refractive Error and/or Accommodative Deficit [26,27]	38.6 (76/197)	Baseline	17.5 (35)	p<0.001
			[13 no/poor compliance with current spectacles, 13 no previous Rx, 6 update Rx]	
		Follow-up	8.5 (17)	
			[14 no/poor compliance; 3 new/updated spectacle Rx not acquired]	
Reduced contrast sensitivity [28]	13.1 (24/183)	Baseline	12.0 (24)	p<0.001
		Follow-up	3.5 (7)	
Reduced distance and/or near acuity [33,34]	8.6 (17/197)	Baseline	5.0 (10)	p = 0.016
		Follow-up	2.0 (4)	
Ocular pathology/anomaly	(18/199)	Baseline	3.0 (6)	p = 0.016
		Follow-up	0 (0)	
Visual field deficit[29]	2.1 (4/187)	Baseline	2.0 (4)	p = 0.250
		Follow-up	1 (2)	
Anomalous eye movement control [35,36]	(20/177)	Baseline	6.5 (13)	p<0.001
		Follow-up	0.5 (1)	
Evidence of visual processing deficits [30,37]	(43/183)	Baseline	21.0 (42)	p<0.001
		Follow-up	6.0 (12)	
TOTAL	61.0 (122/200) with at least one visual deficit	Baseline	45.0 (90)	p<0.001
		Follow-up	18.1 (36)	

*Methods of meeting unmet visual needs included provision of new/updated spectacles to correct refractive deficits, implementation of advice relating to environmental modifications such as increased print/image size and increased contrast, onward referral for treatable ocular and eye movement disorders such as blepharitis or reduced near point of convergence.

Refractive deficits, reduced contrast sensitivity and evidence of higher visual processing deficits were not only the most common presenting deficits, they were also the least well-met visual needs. Younger age and no previous history of eyecare were independently associated with increased odds of having at least one unmet visual need at baseline (Odds ratio 1.12 95% CI 1.04 to 1.21, p = 0.012; Odds ratio 4.44 95% CI 1.38 to 14.29 p = 0.007 respectively). Neither level of learning disability or gender were significantly associated with the presence of an unmet need at baseline (multinomial regression analysis both p>0.05).

#### Reports and actions to address visual deficits and unmet visual needs

Vision Reports were issued for all participants. All reports summarised participants’ visual status and, where an unmet visual need had been identified, reports included advice or actions required to address these needs. Nine reports initiated onward referral to a general medical practitioner or hospital eye services and 35 contained information about new or updated spectacle correction and/or advice to support spectacle compliance. Nineteen (9.5%) participants with a significant refractive error or accommodative deficit were supplied with a first-time (n = 13) or updated (n = 6) spectacle correction. Sixty-five (32.5%) Vision Reports provided advice about environmental and learning material modification to reduce the impact of contrast deficits, visual field defects, impaired vision and/or evidence of difficulties with visual processing. These reports provided examples of appropriate large print/image size, strategies to increase contrast of learning materials, and modifications to seating position at home and in the classroom to maximise access to learning and recreational stimuli [31]. Where evidence of visual processing difficulties was found, strategies to manage these difficulties were given, as recommended by Dutton et al [21,32] and these difficulties were also communicated to the participants’ wider healthcare teams for further investigation, as appropriate.

Where Vision Reports contained actions and strategies for parents or teachers to implement, 80.3% of parents responded to the question probing whether they had made environmental modifications as suggested in the report. Half (50.9%) reported that they had actioned the suggestions in the report. Teaching staff were also asked to report whether modifications to classroom seating and/or learning material had been made as suggested by the Vision Reports they received. Eighty-eight percent reported that modifications had been made to maximise pupil’s visual access to learning and recreational material.

#### How well were visual needs met at follow-up?

Data from the follow-up eye examination and the parent/teacher feedback questionnaires were used to determine how in-school eyecare had influenced the number of participants whose visual deficits were treated, recognised and/or appropriately addressed. Table 5 illustrates the number of participants with significant visual deficits at baseline and the number whose visual needs were unmet (unrecognised and/or unaddressed) at baseline and follow-up. Significantly more visual needs were met at follow-up (NcNemar’s test p<0.001), with the exception of visual field defects (Table 5).

A statistically significant improvement in presenting near visual acuity was recorded for children where refractive deficits present at baseline had been addressed at follow-up.

At follow-up, the number of participants with unmet needs in relation to refractive error significantly decreased but while a notably greater number (regardless of age, level of learning difficulty or gender, multiple logistic regression p>0.05 for all) who had spectacles were compliant, poor compliance with spectacles remained the primary reason for unmet need in this category. Where refractive deficits present at baseline had been addressed at follow-up, participants demonstrated significantly improved near visual acuities (Wilcoxon signed rank z = -2.226, p = 0.013). This was not the case for peers who either had no unmet need in relation to refractive error at baseline or follow-up, or those who retained an unmet need in relation to refractive error from baseline to follow-up.

The primary aim of the study was to assess the impact of in-school eyecare in special educational settings as described by the sector-approved framework [15]. The framework does not explicitly include assessment of crowding deficits or potential visual processing deficits. If crowding and visual processing difficulties are not considered in these analyses, the findings are similar; the number of participants whose visual needs were all met at follow-up (n = 175) is also statistically significantly greater than at baseline (n = 139) (McNemar’s test p<0.001).

#### Did the in-school eyecare and identification of visual deficits affect classroom engagement and behaviours?

Baseline and follow-up classroom observations were completed for 193 participants. Participants whose parents or teachers had received actions or advice to alleviate unmet visual needs identified at baseline, demonstrated significantly less ‘off task’ behaviour at follow-up (paired Wilcoxon rank test p = 0.035), while their peers showed no such change in behaviour (p = 0.261) (Table 6). Pupils in the former group were younger at baseline, but their age did not significantly influence the magnitude of the improvement in off-task behaviour (Pearson correlation coefficient r = -0.087, p = 0.414). While there was also evidence for improvements in other observed behaviours at follow-up, the improvements failed to reach significance. Although more participants were wearing spectacles during classroom observations at follow-up (n = 54), compared with baseline (n = 51), nine with significant refractive deficits which would have impacted on the clarity of their learning material were not wearing spectacles during the observed period.

**Table 6:** Observed classroom behaviours at baseline vs follow-up using paired data from individual participants’ two measurement periods.

	Participants with at least one unmet visual need at baseline (n = 90)			Participants whose visual needs were met at baseline (n = 110)		
Target behaviour	Baseline Count	Follow-up Count	Wilcoxon signed rank test, z (p)	Baseline Count	Follow-up Count	*Wilcoxon signed rank test, z (p)
	Median (IQR)	Median (IQR)		Median (IQR)	Median (IQR)	
Initiates engagement	3 (1–5)	2 (1–4)	-0.039 (0.485)	3 (1–6)	2 (1–5)	-1.632 (0.052)
Teachers’ positive comment	2 (1–5)	2 (0–4)	-0.115 (0.454)	2 (0–5)	1 (0–3)	-2.617 (0.005)
Off task	1 (0–3)	0 (0–2)	***-1*.*817 (0*.*035)***	0 (0–2)	0 (0–1)	-0.641 (0.261)
Not visually engaged with task	0 (0–0)	0 (0–0)	-0.881 (0.189)	0 (0–0)	0 (0–0)	-0.408 (0.342)
Repetitive behaviour	0 (0–1)	0 (0–0)	-1.525 (0.064)	0 (0–0)	0 (0–1)	-1.632 (0.052)

*The Wilcoxon signed rank test was applied to test the null hypothesis that there was no difference in count between baseline and follow-up against the alternative hypothesis of an improvement in behaviour. Negative z values represent an improvement in the following behaviours (off task, not visually engaged, repetitive behaviours) and positive z values represent an improvement in the remainder (initiates engagement, teachers’ positive comment). Only p-values less than 0.05 which are associated with improved scores (the hypothesis under test) should be considered significant in this one-tailed analysis.

## Discussion

The present study demonstrates measurable benefits associated with providing comprehensive eyecare in a special education setting. Although the majority of participants had a previous history of eyecare, nearly half presented with at least one significant visual deficit which was not currently identified or addressed. After undergoing in-school eyecare with dispensing of spectacles (as appropriate) and written reporting of visual status, the number of children with deficits which were not being appropriately addressed or managed reduced. Where presenting visual deficits were identified through the in-school eye examination and advice or action was given to address previously unrecognised or untreated deficits, significant improvements in aspects of children’s visual function (near visual acuity) and classroom engagement (less time spent ‘off-task’) were measured.

Refractive deficits were common in the present study, as expected from previous studies of similar populations [1,9]. While the participants in the present study had a more comprehensive history of previous eyecare than either Das et al (2010) or Woodhouse et al (2012) refractive deficits still constituted a large portion of the unmet visual need identified at baseline, mainly due to unmanaged accommodative deficits and poor compliance with spectacles. The latter is a well acknowledged issue amongst children with special needs and their typically developing peers [1,9,38]. Children with developmental disability have been shown to have a higher prevalence of accommodative deficits than typically developing children [39–41] and these deficits are relatively easy to quantify and manage with refractive correction, including prescription of bifocal spectacles [42,43]. The majority of children with hypo-accommodation in the present study had a history of eyecare; suggesting that more attention needs to be paid to the measurement and optimisation of accommodative accuracy by eyecare professionals. When visual deficits are identified and addressed, the present study demonstrates improvements in visual function, specifically near visual acuity. Conversely, failure to address poor accommodation has implications for quality of near vision and engagement with learning materials. Previous studies reporting visual status in special educational settings have not reported measures of near vision. Given the importance of both near and distance vision in the learning environment, the high success rates for measuring this function and the improvements measured when unmet visual needs are addressed, our data support the inclusion of this functional measure within comprehensive eye examinations for children with special educational needs and in future research studies.

Spectacle compliance in a large study of typically developing school children [38] revealed that approximately 25% of children who had spectacles (and whose vision was reduced without them), failed to routinely bring their spectacles to school. Compliance was also problematic in the present study; 50% of participants with significant refractive deficits and spectacles did not present to the in-school eye examination wearing their spectacles and parents/teachers reported poor or no compliance. Furthermore, compliance was further reduced when participants were observed in class, rather than presenting for an eye examination and it is likely that the former observation is more reflective of habitual behaviour. At follow-up, significantly more children who needed spectacles presented wearing spectacles and more children were wearing their spectacles during classroom observation. Parent/teacher feedback reported the benefit of strategies to encourage spectacle wear and the added benefit of the eyecare professionals dispensing the spectacles in school and regularly being in the school to support spectacle wear. Nonetheless, compliance with spectacles remained a significant issue, being the most common reason for a refractive deficit being unaddressed at follow-up, suggesting that enhanced strategies and support are needed to encourage compliance with spectacle wear amongst children in special educational settings to promote optimal visual and learning outcomes. An ongoing in-school eyecare service with in-school spectacle dispensing component, would allow for further support in compliance issues and provide the opportunity for in-school repair and replacement of spectacles. Frequent repair and replacement is often a feature of early spectacle wear for children and is essential to maintain good compliance and optimise vision.

‘Environmental’ modifications were advised for a large number of children with visual deficits which could not be eliminated by spectacle wear or other ‘treatment’. Despite the majority of participants having a previous history of eyecare, there was little evidence that such deficits and their day-to-day impact had been effectively articulated to teachers or parents, or included in statutory documents defining the child’s need for support. For example, deficits in contrast sensitivity were common and entirely unaddressed or appreciated at baseline. Contrast sensitivity deficits are relatively easy to measure using validated preferential-looking techniques and whilst they are not generally treatable, advice and environmental modifications are straightforward and inexpensive. Parents and teachers reported that the Vision Report issued after the in-school eye examination was valuable and few had previously received such information [24]. Where environmental modifications such as increased contrast, increased print size or change in seating position were advised in the Vision Report, teachers reported a high level of pro-activity in implementing these low-tech modifications and, as a consequence, the number of children whose visual deficits were appropriately addressed in the school environment were increased. Parents appeared to need more support in implementing environmental modifications in the home. The outcomes of the present study support the inclusion of jargon-free reports describing visual strengths and weaknesses and highlighting actions required to address visual deficits, including environmental modifications, as a necessary component of visual assessments of children with special educational needs. Without such reporting, the findings of the present study suggest visual deficits will remain unaddressed, to the detriment of the child’s vision and learning opportunities.

Visual processing deficits, such as those elicited by the Visual Skills Inventory (VSI), are reported to be common amongst children with developmental disability [16,21,44]. The present study used comparisons between crowded and isolated optotype acuities and the VSI to reveal a large number of children with evidence of visual processing deficits, very few of which had previously been investigated or reported to parents or teachers. In the absence of agreed diagnostic or management protocols, where evidence of visual processing deficits was found strategies to address the specific deficit highlighted by the VSI or crowded/isolated acuity measures were provided to parents and teachers. Liaison with local tertiary care providers allowed for onward referral for further investigation as indicated (n = 4). The high number of unrecognised visual processing deficits revealed in the present study underlines the urgent need for the development of agreed protocols for the diagnosis and management of visual processing deficits. While such deficits are not routinely identified through the methods employed in traditional eye examinations, they can have significant impact on daily living activities and behaviour [21,45,46]. The evaluation of visual processing deficits in the present analysis was limited to two relatively straight-forward measures which were considered achievable in the context of the special school setting. The authors acknowledge that additional visual processing deficits [47], which were unexplored and unaddressed in the present study, could restrict the benefit measured from the in-school vision care. Additionally, some authors have suggested that excessive levels of lighting in standard school rooms may exacerbate visual processing deficits [48,49]. The present study did not formally measure lighting levels in the classroom settings, but baseline and follow-up measures were conducted under semi-standardised conditions.

This is the first study to attempt to measure the impact of in-school eyecare on the classroom behaviour of children in special education settings. Previous work has shown that appropriate vision correction for children with learning disability may result in improvements across a wide range of behaviours; for example, improvements in social behaviour, gross and fine motor skills and literacy were noted in two groups of children (aged 0–6 and 7–17 years) whose vision needs were addressed [50]. The outcomes from the present study demonstrate a significant improvement in classroom engagement in one domain; the amount of time spent ‘off-task’ during the ten-minute observation period. The number of times participants were ‘not visually engaged with the task’ or were engaged in ‘repetitive behaviours’ also decreased from baseline to follow-up, but these improvements failed to reach significance. Two behaviours, ‘initiates engagement’ and ‘teacher’s positive comment’, were recorded on fewer occasions at follow-up. These behaviours may indicate that children were working more independently and needing less encouragement or intervention from the teacher, but the difference in counts is not statistically significant. The authors acknowledge the challenges of identifying whether in-school eyecare and identification of previously unrecognised or unaddressed visual deficits has an impact on classroom behaviour, particularly given the heterogenous nature of the participant group in terms of age and learning disability. Careful, repeat measures by a masked observer using previously validated measures of classroom engagement at baseline and follow-up for each participant, was undertaken to limit the impact of these variables. The time interval between baseline and follow-up was also designed to be as brief as possible within the constraints of the school timetable and the research protocol, to limit the effect of normal development on measures. Observations were undertaken in a standardised way but it was not possible to entirely standardise the type of learning activity the participants were engaging in during the timed period, and some variation existed between baseline and follow-up observation activities. Ideally, the same activity would have been set for the observation periods at baseline and follow-up and this may have proved a more sensitive methodology with which to explore improvements in engagement and participation following in-school eyecare. Further evaluation of the impact of in-school eyecare on classroom behaviours though individual case studies is planned.

The participation rate of pupils from the participating school was high and the children and young people involved in the study were representative of the underlying pupil profile. Although nearly 30% required more than one attempt to complete the eye examination, success rates in obtaining formal measurement across a comprehensive range of visual functions was high when given this opportunity, illustrating that provision of in-school eye care in special education settings should yield a high level of success in providing a thorough evaluation of children’s visual status and needs. While the pupil profile of the present school reflects primarily individuals with moderate and severe learning disability, and therefore the outcomes may not be entirely generalisable to pupils with more profound impairments, Donaldson et al (2019) demonstrate that in-school eye examinations can also be successfully applied in schools catering for more pupils with profound and complex needs.

The UK government has identified the need for better access to healthcare for children with special needs and improved cooperation and sharing of information between healthcare and educational services [51–55]. The importance of delivering care in the most appropriate setting, with minimal disruption to education, has also been identified as an important component of paediatric health services [56–57]. Delivering comprehensive eyecare services in special education settings promotes improved access to eyecare, facilitates information flow between health, education and families and causes minimal disruption to children’s education. Evaluation of parent and teacher views on the benefits and drawbacks of in-school eyecare is ongoing. The outcomes from the present analysis demonstrate that not only does in-school eyecare in special education settings address government aspirations in relation to equality of care for children with learning disabilities, it has measurable benefits for their visual status and opportunities to engage with learning material.

### Conclusions

The present study demonstrates measurable benefits to children and young people in a special education setting from undergoing comprehensive in-school eye examinations with on-site spectacle dispensing and jargon-free reporting of outcomes to teachers and parents. Benefits were apparent in both visual and behavioural domains. In-school eyecare services offer an opportunity to improve health and education outcomes for people with learning disability.

## Supporting information

S1 Fig Anonymised data set. (PDF)
