# Correction: In-school eyecare in special education settings has measurable benefits for children’s vision and behaviour

**DOI:** 10.1371/journal.pone.0222300

**Published:** 2019-09-05

**Authors:** S. A. Black, E. L. McConnell, L. McKerr, J. F. McClelland, J. A. Little, K. Dillenburger, A. J. Jackson, P. M. Anketell, K. J. Saunders

There are errors in the Author Contributions. The correct contributions are: Conceptualization: JFM JAL KD KJS. Data curation: SAB ELM LM. Formal analysis: SAB ELM. Funding acquisition: JFM JAL KD KJS. Investigation: SAB ELM LM. Methodology: SAB ELM LM KJS. Project administration: SAB ELM LM. Resources: KJS. Supervision: JFM JAL KD KJS. Visualization: KJS. Writing–original draft: SAB. Writing–review & editing: ELM LM JFM JAL KD AJJ PMA KJS.

The ORCID iDs are missing for the second, third, fourth, fifth, and ninth authors. Please see the authors’ respective ORCID iDs here:

- Author E. L. McConnell’s ORCID iD is: 0000-0002-6180-418X (https://orcid.org/0000-0002-6180-418X).
- Author L. McKerr’s ORCID iD is: 0000-0001-5989-0763 (https://orcid.org/0000-0001-5989-0763).
- Author J. F. McClelland’s ORCID iD is: 0000-0001-9726-3736 (https://orcid.org/0000-0001-9726-3736).
- Author J. A. Little’s ORCID iD is: 0000-0001-5242-8066 (https://orcid.org/0000-0001-5242-8066).
- Author K. J. Saunders’s ORCID iD is: 0000-0002-9289-5731 (https://orcid.org/0000-0002-9289-5731).
