# Supplementary material for: In-school eyecare in special education settings has measurable benefits for children’s vision and behaviour
Source: PLoS One. 2019 Aug 1;14(8):e0220480. doi: 10.1371/journal.pone.0220480 (PMC6675062; doi:10.1371/journal.pone.0220480)
Supplement: S1 Fig — (PDF) [file pone.0220480.s001.pdf]

PxID	Visits	Age	Gender	ParentQ	PrevEyeT	WhereEyesT	Present	TeachparentimplMOD	LD	Autism	CP	Down	CTN	CTD	StereoInit	CS	VAtestD	PRESVA	VAtestN	BINOCN	FocA25	Cyclo	RSER	LSER	ClinicallySE2	Tracking	NPCinit	OcularDEF	Nystagmus	ParEyeprobs	HomeMod	TeachQret	TeachEP	TeachMod
SP001	2	8.42	0	1	1	1	0	0	2	3	0	0	0	0			4	0.200	0		4.00	1	1.00	1.00	0	0	5	11	0	0	0	1	0	0
SP002	2	8.92	1	1	1	1	1	2	2	3	1	0	2	2	0		3	0.200	1	0.400	2.50	1	2.50	2.50	0	0	5	0	0		0	1	0	0
SP003	2	8.42	1	0			0	2	2	3	0	1	0	0	3	33.33	3	0.200	1	0.300	3.33	1	0.50	0.25	0	0	9	0	0			1	0	0
SP004	2	7.67	0	1	1	1	0	1	2	3	0	0	2	2	0	16.67	3	0.400	1	0.600	4.00	1	-0.50	-0.75	0	0	5	11	0	1	1	1	0	0
SP005	2	7.25	0	1	1	1	1	1	2	3	0	0	1	1		33.33	2	0.200	0		4.00	0	2.00	2.00	1	0	0	0	0	1	0	1	0	0
SP006	1	7.42	1	1	1	1	0	1	2	3	0	0	0	0	3	100.00	3	0.100	1	0.200	4.00	1	-0.50	-0.50	0	0	0	0	0	0	0	1	0	0
SP007	2	7.92	1	1	1	1	0	1	2	3	0	1	0	0	3	50.00	3	0.300	1	0.100	3.33	1	0.25	0.25	0	0	0	0	0			1	0	0
SP008	2	7.17	0	1	1	1	0	1	2	3	0	0	0	0	3	50.00	4	0.000	1	0.500	4.00	1	0.50	0.50	0		5	0	0	0	0	1	0	0
SP009	2	5.42	1	1	1	1	1	0	2	3	0	0	2	2	0	16.67	2	0.000	0		2.94	1	0.00	0.00	1	0		0	0	1	0	1	1	1
SP010	2	5.50	1	1	1	1	0	1	3	3	0	1	0	0		16.67	2	0.400	0		4.00	0	0.25	0.25	1	0	0	2	0	0	0	1	2	0
SP011	2	5.42	0	1	1	1	0	2	2	3	0	0	2	2			1	0.920	0		2.00	1	4.50	4.50	1	0		9	1	1	1	1	1	1
SP012	2	5.67	1	1	1	0	0	0	2	1	0	0	0	0			2	1.200	0		4.00	1	0.00	0.00	0	0	9	0	0	0	0	1	0	0
SP013	2	7.08	1	1	1	1	1	2	2	3	0	1	0	0			3	0.300	1	0.400	4.00	1	3.00	3.00	1	0	5	0	0	1	0	1	0	0
SP014	2	9.92	0	1	1	1	0	0	2	3	0	0	0	0		50.00	3	0.200	0		4.00	1	-0.25	-0.25	0			0	0	0	0	1	0	0
SP015	2	6.00	1	1	1	1	0	0	2	3	0	0	0	0		12.00	2	0.200	0		4.00	1	1.00	0.00	0		0	0	0	0	0	1	0	0
SP016	2	6.33	1	1	1	1	0	2	2	1	0	0	0	0	1	50.00	2	0.100	0		4.00	1	1.00	1.00	0		0	0	0	0	0	1	0	0
SP017	2	8.33	1	1	1	1	1	2	8	0	2	2	0	0			3	0.500	0		4.00	1	6.50	6.50	1	0	9	2	0		0	1	0	0
SP018	4	9.67	1	1	1	1	0	1	2	1	0	0	0	1		50.00	2	0.000	0		2.50	1	-0.75	-0.75	1	0	0	11	0	1	0	1	0	0
SP019	2	7.33	1	2	0		0	2	2	1	0	0	0	5		33.33	2	0.100	0		4.00	1	0.25	0.00	0	0	9	0	0			1	0	0
SP020	2	9.83	1	1	1	1	0	1	2	1	0	0	0	0		33.33	2	0.100	0		4.00	1	1.50	1.75	0	0	0	0	0	1	0	1	0	0
SP021	1	15.17	1	1	1	1	1	1	2	3	0	1	0	0	3	50.00	5	0.125	2	0.050	4.00	1	5.00	5.50	1	0	3	0	0	1	0	0		
SP022	2	14.33	0	1	1	2	0	1	2	3	0	0	2	2	1	66.66	5	0.025	2	0.150	3.00	1	1.50	0.25	0	0	16	6	0	0	0	0		
SP023	1	15.92	0	1	0		0	2	2	3	0	0	0	0	3	100.00	5	-0.100	2	0.000	4.00	1	-0.25	0.25	0	0	0	0	0	0	0	1	0	0
SP024	1	9.75	1	1	1	1	0	2	4	1	0	0	0	0	2	100.00	3	-0.100	1	0.000	4.00	1	-0.25	-0.50	0	0	0	0	0	0	0	1	0	0
SP025	1	14.58	1	1	1	1	0	0	2	3	0	1	0	0	3		5	-0.025	2	0.025	4.00	1	1.50	1.50	0	0	8	0	0	1	0	1	0	0
SP026	2	13.67	1	1	1	2	0	1	3	3	0	0	0	1		66.66	5	-0.075	2	0.125	3.00	1	0.25	0.50	0		0	0	0	1	1	0		
SP027	2	10.92	1	1	1	1	0	1	2	3	0	0	0	0		12.50	2	0.200	0		2.50	1	3.50	3.50	1	0	0	11	0	1	0	1	0	0
SP028	3	10.75	1	1	1	1	0	0	4	1	0	0	0	0	2	100.00	5	0.000	2	0.000	4.00	0	0.25	0.50	0	0	9	0	0	0	0	1	0	0
SP029	2	10.58	1	1	0		0	0	4	1	0	0	0	0	1	100.00	3	0.200	1	0.200	4.00	1	2.50	2.25	1	0	0	0	1	1	0	1	0	0
SP030	2	10.83	1	1	1	1	1	0	0	1	0	0	0	0	3	100.00	3	0.100	1	0.100	4.00	1	2.00	2.00	1	0	0	0	0	1	0	1	2	0
SP031	2	16.58	1	1	1	1	0	1	2	3	0	0	0	1		33.33	2	0.100	0		4.00	1	0.00	-0.50	0	0	7	0	0	0	0	0		
SP032	1	16.67	0	0			0	1	2	1	0	0	0	0			2	0.200	0		4.00	1	-1.25	-1.25	0	0	0	0	0			0		
SP033	2	14.33	0	1	1	1	0	1	2	1	0	1	1	1			1	1.200	0		6.67	1	-6.50	-2.75	1	0		9	1	1	1	0		
SP034	1	5.00	1	2	1	2	0	1	2	1	0	0	0	0		33.33	2	0.200	0		2.00	1	1.00	0.50	1	0	0	0	0			0		
SP035	1	5.08	0	1	1	1	0	0	3	3	0	0	0	0			2	0.000	0		4.00	1	0.00	0.00	0	0	0	11	0	0	0	0		
SP036	1	9.25	0	1	1	1	0	2	1	3	1	0	2	2			1		0			1	-3.00	-3.50	1			1	1	1	1	0		
SP037	1	7.92	1	1	1	1	0	2	8	0	2	2	0	0		8.33	2	0.100	0		4.00	1	1.25	1.25	0		9	0	0	0	0	0		
SP038	1	6.42	1	1	1	1	0	1	6	3	0	0	1	1			2	0.300	0		1.85	1	1.25	0.75	0	0	0	0	0	1	0	0		
SP039	1	5.33	0	1	1	1	0	0	1	3	0	0	1	5			1	0.400	0			1	1.50	1.75	1	0	0	4	0	1	1	0		
SP040	1	11.83	1	1	0		0	0	7	1	0	0	0	0	3	66.66	5	-0.075	2	0.025	4.00	1	0.00	0.00	0	0	0	0	0	0	0	1	0	0
SP041	1	4.50	1	1	1	3	0	0	3	1	0	0	0	0	2		2	0.000	0		2.00	1	0.00	0.00	0	0	0	11	0	2	0	0		
SP042	1	12.50	1	1	1	1	0	1	2	3	0	0	3	0			1	1.300	0			1	-0.50	-0.50	0	0		0	0	1	1	0		
SP043	2	13.17	1	1	1	1	0	2	2	1	0	0	0	0	3	100.00	3	0.000	1	0.000	4.00	1	-0.25	0.00	0	0	0	0	0	0	0	1	0	0
SP044	3	10.83	1	1	0		0	0	2	1	0	0	0	0		16.67	2	0.000	0		4.00	1	0.00	0.00	0	0	0	11	0	0	0	1	0	0
SP045	1	17.67	0	1	1	1	1	1	2	3	0	0	0	0	3	100.00	5	0.000	2	0.225	4.00	1	2.00	2.00	0	0	7	0	0	1	1	1	1	0
SP046	1	18.58	0	1	1	2	1	2	2	3	0	0	0	0	3	100.00	5	-0.150	2	0.125	4.00	1	-3.50	-3.00	1	0	6	0	0	0	0	1	1	0
SP047	1	19.42	1	0			0	2	2	3	0	0	0	0	3	50.00	5	0.050	2	0.125	4.00	0	-0.50	-0.50	0	0	0	0	0			1	0	0
SP048	1	19.33	0	1	1	1	1	2	2	3	0	0	1	1	1	100.00	5	0.000	2	0.200	3.00	1	1.50	1.50	1	0	0	0	0	1	0	1	2	0
SP049	1	18.33	1	2	1	2	0	2	2	2	0	0	0	0	2	100.00	5	-0.300	2	0.000	4.00	1	0.00	0.50	0	0	0	0	0			1	0	0
SP050	1	19.25	1	1	1	2	0	0	2	3	0	1	0	0		12.50	2	0.300	0		2.04	1	1.75	1.50	0	0	0	1	0	1	0	1	0	0
SP051	1	17.75	1	1	0		0	2	2	1	0	0	0	0			2		0			1	-2.50	-3.00	0	0		0	0	2	0	1	0	0
SP052	2	19.75	0	0			0	2	2	3	0	1	1	1		66.66	2	0.400	1	0.100	2.63	1	-0.75	-0.75	1		0	0	0			1	0	0
SP053	1	16.58	1	2	1	2	1	2	2	3	0	1	0	0	3	66.66	3	0.200	1	0.300	2.94	1	3.50	3.25	1	0	0	0	0		0	1	0	0
SP054	1	17.08	1	1	1	2	1	2	2	3	0	1	1	1		100.00	5	0.125	2	0.225	2.50	1	3.75	4.50	1		3	5	0	1	0	1	2	1
SP055	1	16.17	1	1	1	2	1	2	2	3	0	0	0	0	3	100.00	5	-0.050	2		4.00	1	-1.00	-0.75	1	0	0	0	0	1	0	1	0	1
SP056	1	14.67	1	1	1	0	0	0	4	1	0	0	0	0	3	100.00	5	-0.175	2	0.000	4.00	1	-0.25	-0.25	0	0	2	0	0	0	0	1	0	0
SP057	1	14.75	1	1	1	0	0	1	3	1	0	0	0	0	2	100.00	5	-0.150	2	0.000	4.00	0	0.25	0.25	0	0	0	0	0	0	0	1	0	0
SP058	1	15.50	1	1	0		0	0	2	1	0	0	0	0		100.00	5	-0.275	2	0.000	4.00	1	-0.25	-0.50	0	0	6	0	0	0	0	1	0	0
SP059	2	15.75	0	1	1	1	1	2	4	3	0	0	0	0	2	16.67	5	0.325	2	0.400	4.00	1	-0.50	-1.00	1	0	9	0	0	1	0	1	1	1
SP060	1	18.83	0	1	1	0	1	2	2	3	0	0	0	0	3	66.66	5	0.150	2		4.00	1	-0.25	-0.75	0	0	0	0	0	0	0	0		
SP061	1	16.08	0	2	1	0	1	2	2	3	0	0	0	0	1	100.00	5	0.000	2	0.125	4.00	1	4.50	4.75	1		9	0	0			0		
SP062	1	15.83	1	1	0		0	0	4	1	0	0	0	0		100.00	5	0.100	2	0.200	4.00	0	-0.75	-0.75	0			11	0	0	0	1	2	0
SP063	1	16.17	1	1	1	2	1	1	4	1	0	0	0	0	3	100.00	5	-0.275	2	0.000	4.00	1	0.50	-3.00	1	0	0	0	0	1	0	1	2	0
SP064	1	15.67	1	1	1	0	0	2	8	0	2	2	0	0	3	100.00	5	-0.075	2	0.025	4.00	1	-1.75	-1.50	0	0	0	0	0	1	0	1	2	
SP065	2	7.00	1	1	1	1	0	2	2	1	0	0	1	1		33.33	2	0.200	0		2.22	1	0.50	2.00	0	0	5	0	0	2	1	0		
SP066	1	14.25	1	1	1	2	1	1	4	3	0	0	0	0	3	50.00	5	-0.075	2	0.000	4.00	1	2.25	1.75	1	0	0	0	0	1	0	1	0	0
SP067	1	15.75	1	2	1	2	0	2	4	3	0	0	0	0	2	100.00	5	-0.300	2	0.025	4.00	1	0.75	0.50	0	0	0	0	0			1	1	0
SP068	1	16.50	1	1	1	2	1	0	4	1	0	0	0	0	3	100.00	5	-0.175	2	0.000	4.00	1	0.25	1.75	1	0	0	0	0		0	1	2	0
SP069	1	15.58	0	1	1	2	1	2	4	3	0	0	0	0	0	33.33	5	-0.050	2	0.025	4.00	1	-1.00	-0.50	0	0	0	0	0	1	0	1	2	0
SP070	1	14.00	0	1	1	1	1	2	4	3	0	0	0	0	3	100.00	5	-0.175	2		4.00	1	2.00	-0.50	0	0	0	1	0	1	1	1	0	0
SP071	1	14.17	1	1	1	2	0	2	2	2	0	0	0	0	3	100.00	5	-0.200	2	0.025	4.00	1	0.75	0.50	0	0	0	0	0	0	0	1	0	0
SP072	1	14.33	1	1	1	0	0	0	4	3	0	0	0	0	3	100.00	5	-0.275	2	0.000	4.00	1	0.00	0.75	0	0	5	0	0	0	0	1	0	0
SP073	1	14.42	1	1	1	2	1	0	2	3	0	0	0	0	0	50.00	5	0.175	2	0.025	3.00	0	-14.00	-12.00	1	0	9	11	1	1	0	1	2	0
SP074	1	14.08	1	1	1	2	0	0	4	1	0	0	0	0	3	100.00	5	-0.250	2	0.000	4.00	1	0.75	0.75	0	0	0	0	0	0	0	1	0	0
SP075	1	13.67	1	0			1	2	4	3	0	0	1	1	1	50.00	5	0.150	2	0.200	4.00	1	5.75	6.00	1		0	0	1			1	2	0
SP076	1	12.83	1	1	1	0	0	0	4	3	0	0	0	0	3	100.00	5	-0.250	2	0.025	4.00	1	0.75	0.75	0	0	0	0	0	0	0	1	0	0
SP077	1	13.42	1	1	1	2	1	0	4	2	0	0	0	0	3	100.00	5	-0.100	2	0.000	4.00	1	4.00	3.75	1	0	0	0	0	1	0	1	2	0
SP078	1	13.83	1	1	1	2	0	2	4	3	0	0	0	0	3	100.00	5	-0.275	2	0.000	4.00	1	0.00	0.25	0	0	5	0	0	0	0	1	0	0
SP079	2	13.75	1	1	1	2	1	0	4	3	0	0	0	0	3	100.00	5	0.075	2	0.150	4.00	1	6.75	6.75	1	0	9	0	0	1	0	1	0	0
SP080	1	13.50	1	1	1	2	1	0	4	1	0	0	0	0	3	100.00	5	-0.025	2	0.025	4.00	1	0.00	0.00	0	0	4	0	0	2	0	1	0	0
SP081	2	13.92	0	1	1	2	1	1	3	3	1	0	2	2	0	100.00	5	0.150	2	0.275	2.50	0	1.00	-0.75	0			0	1	1	0	1	2	0
SP082	1	13.42	0	1	1	2	0	0	4	3	0	0	0	0	3	100.00	5	-0.225	2	0.000	4.00	1	0.25	0.25	0	0	0	0	0	1	0	1	0	0
SP083	1	13.58	1	1	1	2	0	2	3	3	0	0	0	0	3	100.00	5	-0.250	2	0.025	4.00	1	1.00	1.25	0	0	0	0	0	1	0	1	0	0
SP084	2	13.33	1	2	1	2	0	2	4	3	0	0	0	0	3	100.00	5	-0.200	2	0.050	4.00	1	0.75	0.75	0	0	0	0	0			1	2	0
SP085	1	13.50	0	1	1	1	0	2	4	3	0	0	0	0	2	50.00	5	-0.175	2	0.225	4.00	1	-0.25	-0.25	0	0	0	0	0	0	0	1	0	0
SP086	1	13.67	0	1	1	2	0	2	8	0	2	2	2	2	0	50.00	5	0.050	2	0.150	6.25	1	0.50	0.75	0	0	5	0	0	1	0	1	1	0
SP087	2	13.42	0	1	1	2	0	2	3	3	0	0	0	0	2	66.66	5	-0.125	2	0.000	6.25	1	1.50	1.50	0	0	9	0	0	0	0	1	0	1
SP088	1	13.50	0	1	1	1	1	1	4	3	0	0	2	2	0	100.00	5	0.250	2	0.400		0	-2.75	-2.50	1			8	0	1	1	1	1	1
SP089	1	18.58	1	0			1	2	3	3	0	0	2	2	3	100.00	5	0.025	2	0.150	4.00	1	-3.50	-3.25	1	0	8	0	1			1	2	0
SP090	1	17.42	0	1	0		0	0	3	3	0	0	0	0	3	100.00	5	-0.125	2	0.000	4.00	1	0.00	0.25	0	0	0	0	0	2	0	0		
SP091	1	17.67	0	1	1	1	1	2	4	3	0	0	0	0	2	100.00	5	0.025	2		4.00	1	4.25	2.75	1	0	0	0	0	1	0	1	0	0
SP092	1	14.67	1	1	1	2	0	0	4	3	0	0	0	0	3	100.00	5	-0.100	2	0.000	4.55	1	0.50	0.50	0	0	0	0	0	0	0	1	0	0
SP093	1	12.92	1	0			0	1	4	1	0	0	0	0	3	100.00	5	-0.175	2	0.025	4.00	0	-0.25	0.25	0	0	6	0	0			0		
SP094	1	13.83	0	1	1	2	0	2	4	3	0	0	0	0	2	100.00	5	-0.125	2	0.075	4.55	1	0.00	-0.25	0	0	9	0	0	0	0	1	0	0
SP095	1	15.17	0	1	1	0	0	2	4	3	0	0	0	0	2	66.66	5	-0.125	2	0.225	2.85	1	0.75	0.50	0	0	12	0	0	1	0	1	0	0
SP096	1	13.83	1	1	1	0	0	0	4	3	0	0	0	0	3	100.00	5	-0.025	2	0.050	4.76	1	0.00	0.25	0	0	0	0	0	0	0	1	0	0
SP097	1	13.92	0	2	1	0	0	0	0	3	0	0	0	0	3	100.00	5	-0.150	2	0.000	4.00	1	0.75	1.00	0	0	0	0	0			1	0	0
SP098	1	12.75	0	1	1	2	1	1	4	3	0	0	4	4	0	100.00	5	-0.050	2	0.000	3.00	1	3.25	4.50	1	0	0	0	0	1	0	0		
SP099	1	12.92	0	1	1	2	0	1	4	2	0	0	0	0	3	100.00	5	-0.225	2	0.000	4.00	1	1.25	1.00	0	0	0	0	0	0	0	0		
SP100	1	12.50	1	1	0		0	1	4	3	0	0	0	0	3	100.00	5	-0.100	2	0.025	4.00	1	0.00	0.25	0	0	20	0	0	0	0	0		
SP101	1	12.92	1	2	1	1	0	1	2	3	0	0	0	0	2	100.00	5	-0.200	2	0.000	4.00	1	0.75	0.75	0	0	6	0	0			0		

SP102	1	12.58	1	1	1	2	1	1	3	3	0	1	0	0	3	100.00	5	0.175	2	0.125	4.00	1	3.25	3.75	1	1	0	0	0	1	1	0		
SP103	1	12.92	1	1	1	1	0	2	4	1	0	0	0	0	3	100.00	5	-0.175	2	0.000	4.00	0	-0.50	0.00	0	0	0	0	0	1	0	1	0	0
SP104	2	11.75	1	1	1	2	0	2	4	3	0	0	0	0	3	100.00	5	-0.075	2	0.050	2.50	1	0.00	0.00	0	0	0	0	0	0	0	1	0	0
SP105	1	12.33	1	0			0	0	4	2	0	0	0	0	3	100.00	5	-0.250	2	0.000	4.00	1	1.25	1.25	0	0	0	0	0			1	2	0
SP107	2	11.83	1	2	1	2	0	2	3	3	0	0	0	0	3	100.00	5	0.025	2	0.125	4.00	1	2.25	2.50	0	0	6	0	0			1	2	0
SP108	1	12.00	1	2	1	0	0	0	0	3	0	0	0	0	3	100.00	5	-0.250	2	0.000	4.00	1	0.75	0.75	0	0	0	0	0			1	0	0
SP109	2	11.50	0	1	1	2	0	2	3	3	0	0	2	2	0	100.00	5	-0.100	2	0.000	4.00	1	2.00	4.75	1	0		13	0	1	0	1	1	0
SP110	2	11.42	1	1	1	2	0	0	4	1	0	0	0	0	3	100.00	5	0.025	2	0.025	4.00	1	3.25	3.75	1	0	9	0	0			1	1	0
SP111	1	10.58	1	1	0		0	1	4	1	0	0	0	0	3	100.00	5	-0.100	2	0.000	4.00	1	1.00	1.00	0	0	0	0	0	0	0	1	0	0
SP112	1	10.83	1	1	1	2	0	1	4	3	0	0	0	0	3	100.00	5	-0.150	2	0.000	4.00	1	0.75	1.00	0	0	0	0	0	1	0	1	0	0
SP113	1	11.50	1	1	1	2	1	1	2	2	0	0	0	0	2	50.00	5	-0.225	2	0.000	4.00	1	0.50	0.75	0	0	0	0	0	1	0	1	0	0
SP114	1	10.50	1	1	1	2	0	1	4	3	0	0	0	0	3	100.00	5	-0.200	2	0.000	4.00	1	0.25	0.50	0	0	9	0	0	1	0	1	0	0
SP115	1	9.75	0	1	1	1	1	1	2	3	0	0	0	0	1	100.00	5	0.175	2	0.300	2.94	1	5.00	4.50	1		9	0	1	1	0	1	0	0
SP116	2	10.08	1	1	1	1	1	1	4	3	0	0	0	0	0	33.33	5	0.025	2	0.125	4.76	0	-12.75	-9.50	1	1	20	14	0	1	0	1	1	1
SP117	1	9.83	1	1	1	2	1	1	0	3	0	0	0	0	0	50.00	5	-0.075	2	0.000	1.85	1	6.75	7.25	1	0	9	0	0	1	1	1	0	1
SP118	1	10.17	0	1	1	1	0	1	3	3	0	0	0	2	3	100.00	5	-0.100	2	0.025	4.00	1	0.75	0.75	0	0	5	0	0	0	0	1	0	1
SP119	1	9.50	1	2	1	2	1	1	4	3	0	0	0	3	0	100.00	5	0.075	2	0.075	4.00	1	3.50	3.75	1	0	9	0	0			1	2	1
SP120	1	10.17	1	1	0		0	1	4	1	0	0	0	0	2	100.00	5	-0.125	2	0.025	4.00	1	2.25	1.00	0	0	4	0	0	0	0	1	0	1
SP121	1	10.17	1	1	1	3	0	1	4	3	0	0	0	0	3	100.00	5	-0.175	2	0.000	4.00	1	1.25	0.75	0	0	7	0	0	0	0	1	0	1
SP122	2	9.83	1	1	1	1	0	1	2	3	0	0	1	1	2	100.00	5	-0.025	2	0.125	2.22	1	1.25	2.00	1			0	0	1	0	1	0	0
SP123	2	10.33	1	1	1	1	0	1	0	3	1	0	0	0	0	50.00	2	0.000	0		2.50	1	2.50	2.75	0	1		0	0	0	0	1	0	0
SP124	1	10.83	1	2	1	2	0	1	4	1	0	0	0	0	3	100.00	5	-0.150	2	0.025	2.22	0	-0.50	-0.25	0	1	5	0	0			1	0	0
SP125	1	11.25	0	1	1	2	0	1	8	0	2	2	0	0	3	100.00	5	-0.125	2	0.050	4.00	1	0.75	1.00	0	0	0	0	0	1	1	1	0	1
SP126	1	10.58	1	1	1	2	1	1	4	3	0	0	0	0	1	100.00	5	-0.025	2	0.050	2.85	1	3.75	4.50	1	0	9	0	0	1	0	1	0	0
SP127	1	10.50	0	1	1	2	1	1	4	0	2	2	0	0	3	100.00	5	-0.150	2	0.025	4.00	1	3.50	3.50	1	0	0	0	0	1	0	0		
SP128	1	9.92	1	1	1	3	0	1	4	3	0	0	0	0	3	100.00	5	-0.175	2	0.000	4.00	1	1.25	1.25	0	0	0	0	0	0	0	1	0	1
SP129	3	13.17	1	1	1	0	0	1	2	3	0	0	0	0	3	100.00	5	-0.025	2	0.000	4.00	1	-0.25	-0.50	0	0	0	0	0	0	0	1	2	0
SP130	2	13.25	1	1	1	2	1	1	8	0	2	2	0	0	3	50.00	5	-0.050	2		4.00	0	-0.75	-0.50	0	0	0	0	0	1	0	1	0	0
SP131	1	13.08	1	1	1	1	0	0	4	1	0	0	0	0	3	100.00	5	-0.250	2	0.000	4.00	1	0.75	0.75	0		0	0	0	0	0	1	0	0
SP132	1	13.25	1	1	1	3	0	0	4	0	0	0	0	0	3	100.00	5	-0.125	2	0.000	4.00	1	0.75	1.25	0	0	9	0	0		0	1	0	0
SP133	1	12.33	1	1	1	2	1	0	4	3	0	0	0	0	2	100.00	5	-0.125	2	0.000	4.00	1	1.50	2.50	1	0	0	0	0	1	0	1	2	0
SP134	2	11.92	1	1	1	2	1	1	2	3	0	0	1	0	2	100.00	5	0.000	2	0.050	4.00	1	3.50	2.00	1			0	0	1	0	1	1	0
SP135	1	13.08	1	1	1	1	0	0	2	3	0	0	0	0	1	50.00	5	-0.175	2	0.000	4.00	1	0.75	1.00	0	0	8	0	0	0	0	0		
SP136	1	11.50	1	2	1	2	0	2	4	3	0	0	0	0	3	100.00	5	-0.175	2	0.000	4.00	1	2.00	2.25	0	0	0	0	0			1	2	0
SP137	1	11.42	1	1	0		0	2	4	1	0	0	0	0	3	100.00	5	-0.200	2	0.000	4.00	1	0.25	0.50	0	0	0	0	0	0	0	1	0	0
SP138	1	11.50	1	1	1	1	0	0	4	3	0	0	0	0	2	100.00	5	-0.125	2	0.000	4.00	1	0.50	1.00	0	1	0	0	0	0	0	1	0	0
SP139	1	11.75	1	1	1	1	0	2	4	3	0	0	0	0	3	100.00	5	-0.150	2	0.000	3.00	0	-0.25	0.00	0	0	0	0	0	0	0	1	0	0
SP140	1	11.42	0	1	1	2	0	0	2	3	0	1	0	0	3	100.00	5	0.050	2	0.050	3.00	1	0.50	0.25	0	0	0	0	0	0	0	1	2	0
SP141	1	11.83	1	1	1	2	0	2	9	0	2	2	0	0	1	100.00	5	0.100	2	0.000	4.00	1	-1.25	-1.25	0	0	0	0	0	0	0	1	2	0
SP142	1	11.92	0	2	1	2	0	2	2	3	0	0	0	0	3	100.00	5	-0.225	2	0.000	4.00	1	1.50	1.25	0	0	9	0	0			1	2	0
SP143	1	11.75	1	1	1	2	1	1	9	0	2	2	0	0	0	100.00	5	-0.075	2	0.025	4.00	1	6.00	6.25	1	0	10	0	0	0	0	1	2	0
SP144	1	11.67	1	1	1	2	0	1	4	1	0	0	0	0	0	8.33	5	0.000	2	0.050	1.85	1	4.25	1.00	1	0	15	0	0	1	0	1	2	0
SP145	1	10.67	1	1	1	2	1	1	3	1	0	0	0	0	2	100.00	5	0.025	2	0.025	6.67	0	1.75	3.50	1	0	7	0	0	1	0	1	0	0
SP146	1	12.00	1	1	1	1	0	1	4	1	0	0	0	0	3	100.00	5	-0.250	2	0.025	4.00	1	0.50	0.00	0	0	0	0	0	0	0	0		
SP147	1	12.00	1	1	1	2	1	1	4	3	0	0	0	0	2	100.00	5	-0.200	2	0.000	4.00	1	4.50	4.50	1	0	0	0	0	1	0	0		
SP148	1	12.42	0	2	1	2	0	2	5	1	0	0	0	0	3	100.00	5	-0.300	2	0.000	4.00	0	0.75	0.75	0	0	0	0	0			1	0	0
SP149	1	9.25	0	1	1	1	0	1	3	3	0	0	0	0	3	100.00	5	0.000	2	0.025	4.00	0	0.00	0.25	0	0	0	0	0	0	1	0		
SP150	1	9.42	1	1			0	2	4	3	0	0	0	0	3	100.00	5	-0.175	2	0.025	4.00	1	1.00	1.00	0	0	0	0	0	0	0	1	0	0
SP151	1	10.25	1	1	1	0	0	2	4	3	0	0	0	0	3	100.00	5	-0.125	2	0.025	4.00	1	3.25	3.00	0	0	10	0	0	0	0	0		
SP152	1	9.92	1	1	1	2	0	2	4	3	0	0	0	0	3	100.00	5	-0.050	2	0.000	4.76	0	-0.25	-0.25	0	0	0	0	0	0	0	0		
SP153	1	9.50	0	1	1	1	0	1	4	0	2	2	0	0	2	100.00	5	-0.050	2	0.050	4.00	1	2.00	1.75	0	1	8	0	0	0	0	1	0	0
SP154	1	9.42	1	2	1	2	1	0	0	1	0	0	1	1	3	100.00	5	-0.150	2	0.000	4.00	1	1.50	2.25	0	0	0	0	0			0		
SP155	1	9.17	0	1	1	1	0	1	4	3	0	1	0	0	2	50.00	3	0.300	0		4.00	1	1.75	2.00	0	0	8	0	0	0	0	0		
SP156	1	9.25	0	1	1	1	0	2	3	1	0	0	0	0	3	66.66	3	0.000	1	0.000	4.00	1	1.50	0.75	0	0	0	0	0	0	0	0		
SP157	3	9.25	0	1	1	1	0	1	2	1	0	0	0	0		50.00	2	0.000	0		4.00	1	0.00	0.00	0		0	0	0	0	0	1	0	0
SP158	1	7.92	0	0			0	2	4	3	0	0	0	0	3	100.00	3	0.000	1	0.100	4.00	1	0.50	0.25	0	0	0	0	0			0		
SP159	1	6.83	0	1	1	1	0	1	9	1	2	2	0	0		33.33	2	0.000	0		4.00	1	1.00	1.25	0		0	0	0	2	0	1	0	0
SP160	1	7.92	1	1	1	2	1	1	3	3	0	0	0	0	3	100.00	3	0.100	1	0.100	4.00	1	0.50	1.00	0	0	0	0	0	1	0	1	0	1
SP161	1	6.50	1	1	1	1	0	1	4	3	0	0	0	0	3	100.00	5	-0.075	2	0.000	4.00	1	1.75	2.50	0	0	0	0	0	0	0	0		
SP162	1	6.50	0	1	1	1	1	0	3	3	0	0	0	0	1	100.00	6	0.000	1	0.300	2.78	1	-0.25	-0.50	1	0	0	7	0	1	0	1	0	1
SP163	3	7.17	1	1	1	1	0	1	4	3	0	0	0	0	2	100.00	3	0.100	1	0.100	4.00	1	1.75	1.75	0	0	5	4	0	1	0	1	0	0
SP164	1	4.75	0	1	1	1	0	1	2	1	0	0	0	0	3	66.66	3	0.100	1	0.000	4.00	1	1.00	1.00	0	0	0	0	0	1	0	2	1	1
SP165	1	4.58	0	1	1	1	0	1	2	3	0	1	0	0	2	50.00	3	0.300	0		2.13	1	2.25	2.50	0	0	0	0	0	1	0	0		
SP166	2	4.50	1	1	1	1	0	1	4	1	0	0	0	0	0	33.33	2	0.000	0		1.85	1	5.50	6.50	1	0	9	0	0	1	0	2	1	1
SP167	2	6.00	1	1	0		0	1	3	1	0	0	0	0		50.00	2	0.000	0		4.00	1	1.75	2.00	0		0	0	0	2	0	2	1	1
SP168	1	7.25	1	0			1	2	3	1	0	0	0	0		100.00	3	0.000	1	0.100	4.00	1	2.50	3.75	1	0	7	0	0			1	2	1
SP169	1	7.75	1	1	1	2	1	0	3	1	0	0	0	0	3	100.00	5	-0.050	2	0.000	4.00	1	-0.50	0.75	0	0	4	0	0	0	0	1	1	0
SP170	1	8.17	1	1	1	0	1	2	4	3	0	0	0	0	3	100.00	5	-0.025	2	0.000	4.00	1	2.50	3.00	0	0	10	0	0	2	0	1	0	0
SP171	1	8.42	1	1	1	1	0	0	4	3	0	0	0	0	3	100.00	5	-0.175	2	0.000	4.00	1	1.50	1.50	0	0	0	0	0	0	0	1	0	0
SP172	1	7.50	0	1	1	1	0	0	4	1	0	0	0	0	3	100.00	5	-0.125	2	0.000	4.00	1	1.00	1.50	0	0	0	0	0	0	0	1	0	0
SP173	2	8.50	1	1	1	2	0	0	3	1	0	0	0	0	3	100.00	5	-0.300	2	0.000	4.00	1	1.50	2.75	1	0	0	0	0	0	0	1	0	0
SP174	1	7.83	1	1	1	0	0	0	4	3	0	0	0	0	3	100.00	5	-0.100	2	0.000	4.00	1	0.00	0.75	0	0	0	0	0	1	0	1	0	0
SP175	1	5.83	1	2	1	1	0	2	4	1	0	0	0	0		50.00	3	-0.100	1	0.000	4.00	1	-0.25	-0.25	0	0	4	0	0			0		
SP176	1	6.33	1	1	0		0	2	4	1	0	0	0	0		33.33	2	0.200	0		4.00	1	0.50	0.00	0			0	0		0	0		
SP177	1	5.00	1	1	1	1	0	0	2	1	0	0	0	0		33.33	2	0.200	0		4.00	1	3.00	1.75	1		9	0	0	0	0	0		
SP178	1	5.92	1	1	1	1	1	2	8	0	2	2	1	1	0	50.00	2	0.000	0		4.76	1	3.50	4.25	1		0	0	0	1	0	0		
SP179	2	5.25	1	1	1	1	0	0	2	1	0	0	0	0		66.66	2	0.100	0			0	0.00	0.00	0			12	0	0	0	0		
SP180	1	4.75	0	1	1	1	1	2	2	3	0	0	2	2	0	33.33	2	0.100	0		4.00	1	5.75	4.75	1			0	0	1	0	0		
SP182	2	8.50	0	1	1	2	0	2	4	3	0	0	0	0	3	100.00	5	-0.025	2	0.150	4.00	1	1.00	1.25	0	0	0	0	0	2	0	1	1	0
SP183	1	4.92	1	1	1	1	0	0	2	3	1	0	0	0			0		0		2.00	1	2.00	1.75	1			0	0	1	0	0		
SP184	2	5.08	1	2	0		0	1	9	0	2	2	0	0	1	100.00	3	0.100	1	0.100	4.00	1	2.00	1.00	0	0	0	0	0			2	0	0
SP185	1	4.75	1	1			0	1	4	1	0	0	0	0	1	100.00	3	0.100	1	0.100	4.00	1	-0.25	-0.25	0	0	5	0	0	0	0	2	1	0
SP186	1	5.83	1	1	1	1	0	0	4	3	0	0	0	0		100.00	3	0.300	0		4.00	1	1.00	1.00	0	0	0	0	0	0	0	0		
SP187	1	5.25	1	1	0		0	1	3	3	0	0	0	0		50.00	2	0.000	0		4.00	1	1.25	1.75	0		0	0	0	2	0	2	0	0
SP188	1	5.92	1	1	1	1	0	2	2	3	0	1	1	1	0	2.17	2	0.600	0		1.85	1	5.25	5.00	1	0		0	0	1	1	0		
SP189	1	5.33	1	1	0		0	2	2	1	0	0	0	0		50.00	2	0.000	0		2.50	1	1.00	0.00	0	0	9	1	0	0	0	0		
SP190	1	5.58	0	1	1	1	1	0	4	3	0	0	1	1		100.00	5	0.250	2	0.325	3.00	1	6.00	4.00	1	0	6	0	0	1	0	1	0	0
SP191	1	5.67	1	1	1	1	0	2	4	1	0	0	0	0	3	100.00	3	0.000	1	0.200	4.00	1	-0.50	-0.50	0	0	0	0	0	0	0	1	0	0
SP192	2	6.58	0	1	1	1	1	1	3	1	0	0	2	2	0	16.67	5	0.475	2	0.150	4.55	1	0.75	1.00	0			10	0	1	0	1	0	1
SP193	1	5.75	1	2	1	1	0	1	2	1	0	0	0	0	3	100.00	5	-0.025	2	0.025	4.00	1	0.50	1.00	0	1	6	0	0		0	1	0	0
SP194	1	3.75	1	1	0		0	1	4	3	0	0	0	0	1	66.66	2	0.000	0		4.00	1	0.25	-0.25	0	0	5	0	0	2	1	1	2	0
SP195	1	3.58	1	1	0		0	1	9	0	2	2	0	0		66.66	2	0.000	0		4.00	1	1.50	1.50	0	0	0	11	0	0	0	1	0	0
SP196	1	4.50	0	1	1	1	0	1	9	0	2	2	0	0	3	100.00	3	0.100	1	0.400	4.00	1	0.75	0.25	0	0	0	0	0	1	1	1	0	0
SP197	2	4.17	0	1	1	1	1	1	4	3	0	0	1	1	0	50.00	4	0.300	0		4.55	1	3.25	5.00	1			0	0	1	1	1	0	0
SP198	2	4.08	1	1	1	1	0	1	2	1	0	0	0	0		33.33	2	0.100	0		4.00	1	0.25	0.00	0			0	0	1	0	1	0	0
SP199	1	4.33	1	1	1	1	0	1	3	3	0	1	0	0		16.67	2	0.200	0		2.85	1	-5.50	-3.00	1		9	3	0	1		1	2	
SP200	1	4.08	1	1	1	1	0	1	2	1	0	0	2	2		33.33	2	0.000	0		2.70	1	1.50	1.50	0			0	0	1		1	2	1
SP201	1	3.92	1	1	1	0	0	1	2	1	0	0	0	0		66.66	2	0.000	0		4.00	1	0.50	0.50	0	0	7	0	0	2	0	1	0	0
SP202	2	5.25	1	1	1	1	1	1	3	3	0	1	0	0		33.33	2	0.200	0		2.85	1	9.25	9.75	1	1	0	0	0	1	0	2	1	1

ActAdv	TeacherModsImplemented	ParentReprotHomeModsImplementedTwoGroups	FupPresGls	FupCTD	FupCTN	FupNPC	FupCont	FupDVTest	FupPVAD	FupNVTest	FupPVAN	FupRSER	FupLSER	STEREOFUP	FocA25FUP	DEFDVNV	DEFREFACC	DefCS	DEFVF	DEFOM	DefOcular	DEFSQUINT	DEFcrowdCVI	TotDEF	COMBIrefEACC	REDCONTUMN	BVUMN	ODUMN2
1		0	0	0	0			4	0.400			0.50	0.50		4.00	0	0		0	0	0	0	1	1	0	0	0	0
1			1	2	0		50.00	3	0.200	1	0.400	2.00	2.25	0	2.50	1	1		1	0	0	1	0	4	1	0	0	0
1			0	0	0	0	33.30	5	0.200	1	0.400	-1.25	-1.00	2	4.00	0	0	1	0	0	0	0	0	1	0	1	0	0
0	1	0	0	2	2	6	50.00	3	0.400	1	0.400	-1.00	-0.75		4.76	1	0	0	0	0	0	1	0	2	0	0	0	0
1	1	1	1	1	1			1	0.200			2.50	2.50		4.00	0	1	1	0	0	0	1	1	4	0	1	0	0
0	1		0	0	0			3	0.100	1	-0.100	0.00	-0.25	3	4.00	0	0	0	0	0	0	0	0	0	0	0	0	0
0	1	0	0	0	0	2		3	0.200	1	0.100	0.75	0.75		3.00	0	0	0	0	0	0	0	0	0	0	0	0	0
1	1	1	0	0	0			4	0.100	1		1.00	1.00		4.00	1	0	0	0	0	0	0	1	2	0	0	0	0
1		0	1	2	2	6	50.00	2	0.200			-1.50	-1.75		3.00	0	1	1	0		0	1	0	3	0	1	0	0
1		1	0	0	0		12.50	2	0.400			0.75	0.50	1	2.00	1	1	1	0	0	1	0	0	4	1	1	0	0
1			0	1	1			1	0.800						2.00	1	1		0		1	1	0	4	1	0	0	1
0		0	0	0	0			2				0.50	0.50		4.00	1	0		0	0	0	0	0	1	0	0	0	0
1			1	0	0	0	50.00	3	0.300	1	0.400	2.50	3.00	2	4.00	1	1		0	0	0	0	0	2	0	0	0	0
0		0	0	0	0			4	0.100	1	0.300	-0.75	-0.75		4.00	0	0	0	0		0	0	0	0	0	0	0	0
1		0	0	0	0		12.50	2	0.100			0.50	0.50		4.00	0	0	1	0	0	0	0	0	1	0	1	0	0
0			0	0	0			2	0.000			0.50	0.50	2	3.00	0	0	0	0	0	0	0	0	0	0	0	0	0
1			1	0	0		16.70	3	0.600	1	0.300	6.25	6.50	2	4.00	1	1		0	0	1	0	0	3	0	0	0	0
1		1	0	1	1			2	0.100			-0.75	-1.00		4.76	0	1	0	0	0	0	1	0	2	1	0	0	0
0			0	0	0	10		2	0.200			0.25	0.00	1		0	0	0	0	0	0	0	0	0	0	0	0	0
1		1	0	0	0			2	0.100			0.50	1.50	3	2.50	0	0	1	0	0	0	0	0	1	0	1	0	0
0	1	0	1	0	0			5	0.100	2	0.050	5.00	6.50		4.00	0	1	0	0	0	0	0	0	1	0	0	0	0
1	1	0	0	2	2	15		5	0.125	2	0.150	0.75	0.50		4.00	0	0	0	0	1	1	1	0	3	0	0	1	1
0			0	0	0			5	0.100	2	0.000	0.50	0.00		4.00	0	0	0	0	0	0	0	0	0	0	0	0	0
0			0	0	0			3	0.000			0.00	-0.25	1	4.00	0	0	0	0	0	0	0	0	0	0	0	0	0
0		0	0	0	0		66.70	5	-0.025	2	0.000	1.00	1.00		3.00	0	0		0	0	0	0	0	0	0	0	0	0
1	1	0	0	1	1		66.66	5	0.025			0.50	0.00		4.00	0	0	0	0	1	0	1	1	3	0	0	0	0
1		1	1		2			2	0.100			3.50	3.50		4.00	0	1	1	0	0	0	0	0	2	1	1	0	0
0		0	0	0	0	10		5	-0.200	2	0.000	1.00	0.75		4.00	0	0	0	0	0	0	0	0	0	0	0	0	0
1		0	1	0	0			3	0.200			2.25	2.25	3	4.00	0	1	0	0	0	0	0	0	1	1	0	0	0
0		0	1	0	0			3	0.000			1.75	2.50		3.00	0	1	0	0	0	0	0	0	1	0	0	0	0
1	1	0	0	1	0		33.33	2	0.100			-1.00	-1.25		4.00	0	0	1	0	0	0	1	0	2	0	1	0	0
0	1		0	0	0			1	0.500			-0.50	-0.25		4.00	0	0		0	0	0	0	0	0	0	0	0	0
1	1	0	0					1	0.300						2.50	1	1		0		1	1	0	4	1	0	0	0
1		1	1	0	0			2	0.000			1.00	-0.25		2.00	0	1	1	0	0	0	0	0	2	1	1	0	0
0		0	0	0	0		100.00	2	0.000			0.75	0.75		4.00	0	0		0	0	0	0	0	0	0	0	0	0
1			1					0				-3.75	-2.75				1		0		1	1	0	3	1	0	0	0
1			0	0	0			2	0.200			0.75	0.75		4.00	0	0	1	0	0	0	0	0	1	0	1	0	0
0		1	0	1	1			2	0.200			2.00	1.50		2.50	0	1		0	0	0	1	0	2	0	0	0	0
1		0	0				33.30	1	0.400			2.00	2.25		2.50	1	1		0	0	1	1	0	4	1	0	0	0
0		0	0	0	0			5	-0.150			0.50	0.25		4.00	0	0	0	0	0	0	0	0	0	0	0	0	0
0		0	0	0	0			2				1.00	2.75			0	1		0	0	0	0	0	1	0	0	0	0
1	1		0	2	2			1	1.200			0.75	0.75		2.50	1	0		1		0	1	0	3	0	0	0	0
1			0	0	0	2	100.00	3	-0.100			0.00	0.50		4.00	0	0	0	0	0	0	0	1	1	0	0	0	0
0		0	0				66.70	2	0.000			-2.00	-2.00		4.00	0	0	0	0	0	0	0	0	0	0	0	0	0
0		1	1	0	0		100.00	5	-0.150	2	0.050	2.75	2.00		4.00	0	0	0	0	0	0	0	0	0	0	0	0	0
0			1	0	0			5	-0.125	2	0.000	-4.00	-4.25		4.00	0	1	0	0	0	0	0	0	1	0	0	0	0
0			0	0	2		50.00	5	-0.050	2	0.050	-0.50	-0.50		4.00	0	0	0	0	0	0	0	0	0	0	0	0	0
0			1	0	1			5	-0.075	2	0.025	1.75	1.50		4.00	0	1	0	0	0	0	1	0	2	0	0	0	0
0			0	0	0			5	-0.200	2	0.025	0.00	-0.25		4.00	0	0	0	0	0	0	0	0	0	0	0	0	0
1		0	0				12.50	2	0.600			1.00	1.50		2.00	0	1	1	0	0	1	0	0	3	1	1	0	1
0			0	0	0	0		2				-1.50	-1.75		4.76		0		0		0	0	0	0	0	0	0	0
1			0	1	1			2	0.100			-1.25	-1.00		2.70	1	1	0	0	0	0	1	0	3	1	0	0	0
0			1	0	0		66.66	3	0.200	1	0.200	3.25	3.00		3.00	0	1	0	0	0	0	0	0	1	0	0	0	0
1			1	1	1			5	0.125	2	0.225	4.25	5.00		2.85	0	1	0	0	0	1	1	0	3	1	0	0	0
0			0	0	0		100.00	5	0.100	2	0.125	0.25	-0.25		3.00	0	1	0	0	0	0	0	0	1	0	0	0	0
0		0	0	0	0		100.00	5	-0.100	2	0.000	0.25	-0.25		4.00	0	0	0	0	0	0	0	0	0	0	0	0	0
1		1	0	0	0			5	-0.250	2	0.000	-0.25	0.25		4.00	0	0	0	0	0	0	0	1	1	0	0	0	0
0		0	0	0	0			5	-0.250	2	0.025	0.25	-0.50	1	4.00	0	0	0	0	0	0	0	0	0	0	0	0	0
1			1	0	0		100.00	5	-0.025	2	0.200	-0.50	-0.75	2	4.00	1	1	1	0	0	0	0	1	4	0	1	0	0
0			0	0	0			5	0.150	2	0.100	-0.50	-1.50		4.00	0	0	0	0	0	0	0	0	0	0	0	0	0
0			1	0	0			5	0.025	2	0.000	4.25	4.00		4.00	0	1	0	0	0	0	0	0	1	0	0	0	0
0		0	0					0								0	0	0	0		0	0	0	0	0	0	0	0
0		1	0	0	0			5	0.100	2	0.075	0.50	-3.50		4.00	0	1	0	0	0	0	0	0	1	0	0	0	0
0			0	0	0		100.00	5	-0.025	2	0.000	-1.00	-0.75		4.00	0	0	0	0	0	0	0	0	0	0	0	0	0
1																0	1	1	0	0	0	1	0	3	1	1	0	0
0		1	1	0	0			5	-0.150	2	0.025	2.75	3.00	3	4.00	0	1	0	0	0	0	0	0	1	0	0	0	0
0			0	0	0			5	-0.250	2	0.000	0.25	0.00		4.00	0	0	0	0	0	0	0	0	0	0	0	0	0
1		0	1	0	0			5	-0.250	2	0.000	-0.25	0.75		3.00	0	1	0	0	0	0	0	1	2	0	0	0	0
0			1	0	0			5	0.000	2	0.125	-2.50	-1.00		4.00	0	0	0	0	0	0	0	0	0	0	0	0	0
0			0	0	0			5	-0.150	2	0.000	1.25	-2.50		3.00	0	0	0	0	0	1	0	0	1	0	0	0	0
0			0	0	0			5	-0.250	2	0.000	1.00	0.75		4.00	0	0	0	0	0	0	0	0	0	0	0	0	0
1		0	0	0	0			5	-0.250	2	0.025	1.00	0.75		4.00	0	0	0	0	0	0	0	1	1	0	0	0	0
0		0	1	0	0			5	0.175	2	0.125	-13.25	-14.00	0	4.00	0	1	0	0	0	0	0	0	1	0	0	0	0
0		0	0	0	0			5	-0.175	2	0.000	-0.25	-0.25		4.00	0	0	0	0	0	0	0	0	0	0	0	0	0
0			1	1	1			5	0.250	2	0.075	5.75	5.75		4.00	0	1	0	0	0	0	1	0	2	0	0	0	0
0		0	0	0	0			5	-0.225	2	0.000	0.50	0.25		4.00	0	0	0	0	0	0	0	0	0	0	0	0	0
0		0	1	0	0			5	-0.100	2	0.000	4.25	4.00		2.78	0	1	0	0	0	0	0	0	1	0	0	0	0
0			0	0	0		100.00	5	-0.275	2	0.000	0.00	0.00		4.00	0	0	0	0	0	0	0	0	0	0	0	0	0
0		0	1	0	0			5	0.125	2	0.175	6.00	7.50		3.00	0	1	0	0	0	0	0	0	1	0	0	0	0
0		0	1				100.00	5	-0.150	2	0.000	0.00	-0.25		4.00	0	0	0	0	0	0	0	0	0	0	0	0	0
1		1	1	2	2		100.00	5	0.075	2	0.025	0.75	-0.50		2.50	0	1	0	0		0	1	0	2	1	0	0	0
0		0	0	0	0			5	-0.250	2	0.000	0.50	0.00		4.00	0	0	0	0	0	0	0	0	0	0	0	0	0
0			0	0	0			5	-0.225	2	0.000	0.50	0.50		4.00	0	0	0	0	0	0	0	0	0	0	0	0	0
0			0	0	0			5	-0.175	2	0.000	2.00	1.50		3.00	0	0	0	0	0	0	0	0	0	0	0	0	0
0			0	0	0			5	-0.125	2	0.000	0.50	0.50		4.00	0	0	0	0	0	0	0	0	0	0	0	0	0
0			0	2	2			5	0.075	2	0.150	-0.50	-0.25		4.76	0	0	0	0	0	0	1	0	1	0	0	0	0
1			0	0	0		100.00	5	-0.150	2	0.050	0.75	0.25		4.00	0	0	0	0	0	0	0	1	1	0	0	0	0
1		1	1	2	2		100.00	5	0.350	2	0.050	-3.00	-2.50			1	1	0	0		1	1	1	5	0	0	0	0
1			1	2	2			5	-0.050	2	0.150	-4.25	-3.75		4.00	0	1	0	0	0	0	1	0	2	1	0	0	0
0		0	0	0	0			5	-0.275	2	0.000	0.75	0.25		4.00	0	0	0	0	0	0	0	0	0	0	0	0	0
0			1	0	0			5	-0.100	2	0.000	2.50	1.75		4.00	0	1	0	0	0	0	0	0	1	0	0	0	0
0		0	0	0	0			5	0.200	2	0.000	0.75	1.00		4.00	0	0	0	0	0	0	0	0	0	0	0	0	0
0		1	0	0	0			5	-0.200	2	0.000	0.25	0.00		4.00	0	0	0	0	0	0	0	0	0	0	0	0	0
0			0	0	0			5	-0.200	2	0.025	0.00	0.25		4.00	0	0	0	0	0	0	0	0	0	0	0	0	0
1			0	0	0	15	66.66	5	-0.200	2	0.075	0.25	0.50		4.00	0	1	0	0	1	0	0	0	2	1	0	1	0
0		0	0	0	0		100.00	5	-0.025	2	0.050	0.25	-0.25		4.00	0	0	0	0	0	0	0	0	0	0	0	0	0
0		0	0	0	0			5	-0.250	2	0.000	0.25	0.50		4.00	0	0	0	0	0	0	0	0	0	0	0	0	0
1	1		1	0	4			5	0.000	2	0.000	3.25	4.00		2.32	0	1	0	0	0	0	1	0	2	1	0	0	0
0	1		0	0	0			5	-0.175	2	0.000	0.75	0.75		4.00	0	0	0	0	0	0	0	0	0	0	0	0	0
1	1		0	0	0	20		5	-0.200	2	0.025	0.00	0.00	3	4.00	0	0	0	0	1	0	0	0	1	0	0	1	0
0	1		0	0	0			5	-0.200	2	0.025	0.25	0.25		2.85	0	0	0	0	0	0	0	0	0	0	0	0	0

1	1	1	1	0	0		100.00	5	0.175	2	0.025	2.50	3.25		4.00	0	1	0	0	1	0	0	1	3	0	0	0	0
1			0	2	0	0	100.00	5	-0.225	2	0.000	-0.75	-0.75		4.54	0	0	0	0	1	0	1	1	3	0	0	0	0
1			0	0	0			5	0.000	2	0.025	-0.75	-0.75		2.74	0	1	0	0	0	0	0	0	1	1	0	0	0
0		0	0	0	0	0		5	-0.200	2	0.025	0.00	0.25		4.00	0	0	0	0	0	0	0	0	0	0	0	0	0
0			0	0	0			5	0.075	2	0.075	1.75	2.25		2.08	0	0	0	0	0	0	0	0	0	0	0	0	0
0		0	0	0	0			5	-0.275	2	0.000	0.25	0.00		4.00	0	0	0	0	0	0	0	0	0	0	0	0	0
1			0	0	2	30		5	-0.125	2	0.025	0.75	3.75		1.85	0	1	0	0		1	1	0	3	0	0	0	1
1		0	0	0	0			5	-0.025	2	0.000	1.75	2.50		2.27	0	1	0	0	0	0	0	0	1	1	0	0	0
1	1	0	0	0	0			5	-0.150	2	0.000	0.75	0.75		4.00	0	0	0	0	0	0	0	1	1	0	0	0	0
1	1	0	0	0	0			5	-0.150	2	0.175	0.50	0.25		4.00	0	0	0	0	0	0	0	1	1	0	0	0	0
1	1	1	0	0	0			5	-0.025	2	0.000	-0.50	-0.50		4.00	0	0	0	0	0	0	0	1	1	0	0	0	0
1	1	1	0	0	0			5	-0.175	2	0.125	-0.50	-0.25		4.00	0	0	0	0	0	0	0	1	1	0	0	0	0
0	1	0	1	0	0			5	0.125	2	0.125	1.50	3.00	2	4.00	0	1	0	0	0	0	0	0	1	0	0	0	0
1	1		1	0	0	15		5		2	0.000	-12.50	-9.50	0	4.00	0	1	1	0	1	1	0	1	5	0	1	1	1
1	1		1	0	0			5	-0.075	2	0.000	5.25	5.75		2.63	0	1	0	0	0	0	0	1	2	0	0	0	0
1	1	0	0	0	0			5	-0.175	2	0.000	1.25	1.25		4.00	0	0	0	0	1	0	1	1	3	0	0	0	0
0	1		1	3	2			5	0.075	2	0.025	3.25	3.25		2.85	0	1	0	0	1	0	1	0	3	0	0	0	0
0	1		0	0	0			5	-0.100	2	0.025	1.25	0.25		4.00	0	0	0	0	0	0	0	0	0	0	0	0	0
0	1		0	0	0			5	-0.250	2	0.000	0.25	0.25		4.00	0	0	0	0	1	0	0	0	1	0	0	0	0
1	1	1	0	1	0		100.00	5	0.000	2	0.050	1.25	1.00		2.32	0	1	0	0		0	1	0	2	1	0	0	0
1	1		1	0	0		50.00	3	0.200	1	0.500	2.50	2.75		4.00	0	1	0	1	1	0	0	1	4	1	0	1	0
1	1		0	0	0		100.00	5	-0.250	2	0.000	-0.75	-0.50		4.00	0	1	0	0	1	0	0	0	2	1	0	1	0
1	1		0	0	0			5	-0.200	2	0.000	0.00	0.50		4.00	0	0	0	0	1	0	0	1	2	0	0	0	0
1	1	0	1	0	0			5	-0.025	2	0.025	2.75	3.50		4.00	0	1	0	0	0	0	0	1	2	1	0	0	0
1	1		1	0	0			5	-0.150	2	0.000	3.00	2.25		4.00	0	1	0	0	0	0	0	1	2	0	0	0	0
0	1	0	0	0	0			5	-0.275	2	0.000	0.50	0.50		4.00	0	0	0	0	0	0	0	0	0	0	0	0	0
0	1		0	0	0			5	0.050	2	0.075	-0.75	-0.50		4.00	0	0	0	0	0	0	0	0	0	0	0	0	0
1		1	0	0	0			5	-0.025	2	0.050	0.00	-0.50		4.00	0	0	0	0	0	0	0	1	1	0	0	0	0
1		0	0	0	0			5	-0.275	2	0.000	-0.25	-0.50		4.00	0	0	0	0	0	0	0	1	1	0	0	0	0
0		0	0	0	0	8		5	-0.250	2	0.025	0.25	0.50		4.00	0	0	0	0	0	0	0	0	0	0	0	0	0
1		0	1	0	0			5	0.000	2	0.025	0.75	1.75	2	5.26	0	1	0	0	0	0	0	1	2	0	0	0	0
0	1	0	1	0	0			5	0.025	2	0.075	2.75	1.50		2.63	0	1	0	0		0	1	0	2	0	0	0	0
0		0	0	0	0			5	-0.025	2	0.050	0.25			4.00	0	0	0	0	0	0	0	0	0	0	0	0	0
0			0	0	0		100.00	5	-0.150	2	0.000	2.00	1.75		4.00	0	0	0	0	0	0	0	0	0	0	0	0	0
0			0	0	0			5	-0.175	2	0.000	-0.50	-0.50		4.00	0	0	0	0	0	0	0	0	0	0	0	0	0
0		0	0	0	0			5	-0.175	2	0.000	0.25	0.50	2	4.00	0	0	0	0	1	0	0	0	1	0	0	1	0
1			0	0	0			5	-0.200	2	0.000	-0.25	-0.25		4.00	0	0	0	0	0	0	0	1	1	0	0	0	0
0		0	0	0	0			5	0.050	2	0.100	-0.25	-0.25		4.00	0	0	0	0	0	0	0	0	0	0	0	0	0
0			0	0	0			5	-0.100	2	0.025	-1.25	-1.50		4.00	0	0	0	0	0	0	0	0	0	0	0	0	0
0			0	0	0			5	-0.175	2	0.000	0.75	0.50		3.00	0	0	0	0	0	0	0	0	0	0	0	0	0
1		1	1	2	2			5	-0.050	2	0.000	6.00	5.25		4.00	0	1	0	0	1	0	1	0	3	0	0	1	0
1		1	0	0	0			5	0.450	2	0.400	2.50	0.75		1.85	0	1	1	0	0	0	0	1	3	1	1	0	0
1	1	1	1	0	0			5	-0.125	2	0.000	2.00	2.50	3	4.00	0	1	0	0	0	0	0	1	2	1	0	0	0
1	1	1	0	0	0			5	-0.250	2	0.025	0.00	-0.50		4.00	0	0	0	0	0	0	0	1	1	0	0	0	0
0	1		1	0	0			5	-0.200	2	0.000	5.00	5.00		4.00	0	1	0	0	0	0	0	0	1	0	0	0	0
0			0	0	0		100.00	5	-0.225	2	0.000	0.50	0.50		4.00	0	0	0	0	0	0	0	0	0	0	0	0	0
0	1	0	0	0	0			5	-0.125	2	0.000	0.50	0.25		4.00	0	0	0	0	0	0	0	0	0	0	0	0	0
0			0	0	0			5	-0.125	2	0.025	-0.25	0.25		4.00	0	0	0	0	0	0	0	0	0	0	0	0	0
1			0	0	0			5	-0.225	2	0.000	2.75	3.00		2.86	0	0	0	0	1	0	0	0	1	0	0	1	0
0			0	0	0			5	-0.150	2	0.025	-0.25	-0.25		2.50	0	0	0	0	0	0	0	0	0	0	0	0	0
1	1	0	0	0	0			5	-0.050	2	0.000	1.25	1.00		4.00	0	0	0	0	1	0	0	0	1	0	0	1	0
0		0	1	0	1			5	-0.200	2	0.050	1.00	1.50		4.00	0	0	0	0	0	0	1	0	1	0	0	0	0
0		1	0	0	0		50.00	4	0.100	1	0.100	1.00	0.75		4.00	0	0	0	0	0	0	0	0	0	0	0	0	0
0			0	0	0			3	0.000	1	0.000	0.50	0.50		4.00	0	0	0	0	0	0	0	0	0	0	0	0	0
0	1	0	0	0	0			2	0.000					3	4.00	0	0	0	0	0	0	0	0	0	0	0	0	0
0			0	0	0			3	0.000	1	0.100	0.50	0.50		4.00	0	0	0	0	0	0	0	0	0	0	0	0	0
1	1	0	0	0	0		33.33	2	0.200			0.00	0.00		4.00	0	0	1	0	0	0	0	1	2	0	1	0	0
1		1	0	0	0			3	0.100	1	-0.100	0.75	0.50	3	4.00	0	0	0	0	0	0	0	1	1	0	0	0	0
1		1	0	0	0			5	-0.075	2	0.000	0.75	1.50		4.00	0	0	0	0	0	0	0	1	1	0	0	0	0
1		0	1	0	0			4	0.100	1	0.200	-0.75	-1.25		4.00	0	1	0	0	0	1	0	0	2	1	0	0	0
1		1	0	0	0			3	0.100	1	0.100	0.25	0.50		4.00	0	0	0	0	0	1	0	1	2	0	0	0	0
1	1	1	0	0	0			3	0.100	1	0.100	1.25	1.00		4.00	0	0	0	0	0	0	0	1	1	0	0	0	0
1		1	1													0	1	0	0	0	0	0	1	2	1	0	0	0
1	1	1	1	0	0		33.33	2	0.200			5.75	6.50			0	1	1	0	0	0	0	1	3	1	1	0	0
0	1	0	0	0	0			2	0.000						4.00	0	0	0	0	0	0	0	0	0	0	0	0	0
0			1	0	0			3	0.000	1	0.100	2.25	3.00		4.00	0	1	0	0	0	0	0	0	1	0	0	0	0
0		0	0	0	0			5	-0.050	2	0.000	-0.25	0.50		4.00	0	0	0	0	0	0	0	0	0	0	0	0	0
1			0	0	0			5	-0.125	2	0.000	1.00	1.25		4.00	0	0	0	0	1	0	0	0	1	0	0	1	0
0		0	0	0	0			5	-0.125	2	0.000	0.50	0.50		4.00	0	0	0	0	0	0	0	0	0	0	0	0	0
1		0	0	0	0			5	-0.200	2	0.000	0.50	0.50		4.00	0	0	0	0	0	0	0	1	1	0	0	0	0
0		0	0	0	0			5	-0.175	2	0.000	-0.25	2.25		1.85	0	1	0	0	0	0	0	0	1	0	0	0	0
0		0	0	0	0			3	-0.100	1	0.000	0.00	0.25		4.00	0	0	0	0	0	0	0	0	0	0	0	0	0
0			0	0	0			3	-0.100	2	-0.100	0.50	0.25	3	4.00	0	0	0	0	0	0	0	0	0	0	0	0	0
0			0	0	0		50.00	2	0.100							0	0	0	0		0	0	0	0	0	0	0	0
1		0	0	0	0			2	0.300							0	1	1	0	0	0	0	0	2	1	1	0	0
0			1	1	1			2	0.000			3.00			4.00	0	1	0	0	0	0	1	0	2	0	0	0	0
0		0	0	0	0		50.00	2	0.200			1.00	1.50		4.00	0	0	0	0		0	0	0	0	0	0	0	0
1			1	2	2			2	0.000			4.75	4.25		4.00	0	1	1	0		0	1	0	3	0	1	0	0
0			1	0	0			5	-0.025	2	0.050	1.00	1.00	3	4.00	0	0	0	0	0	0	0	0	0	0	0	0	0
1		0	1	0	0			2	0.200						2.50		1		1		0	0	0	2	1	0	0	0
1	1		0	0	0			3	0.100	1	0.000	0.25	0.50		4.00	0	0	0	0	0	0	0	1	1	0	0	0	0
0	1	0	0	0	0			3	0.100	1	0.100	-0.25	-0.25	3	4.00	0	0	0	0	0	0	0	0	0	0	0	0	0
0		0	0	0	0			3	0.000	1	0.200	0.25	-0.25	3	4.00	0	0	0	0	0	0	0	0	0	0	0	0	0
0	1		1					2	0.000						4.00	0	0	0	0	0	0	0	0	0	0	0	0	0
1			0	1	1			1	0.600							1	1	1	0		0	1	1	5	1	1	0	0
0			0	0	0			2	0.100				0.50		2.50	0	1	0	0	0	1	0	0	2	0	0	0	0
0		0	1	0	0		100.00	5	0.000	2	0.175	5.00	5.25		3.00	0	1	0	0	0	0	1	0	2	0	0	0	0
0			0	0	0			3	-0.100	1	0.100	0.00	-0.25		4.00	0	0	0	0	0	0	0	0	0	0	0	0	0
1	1		1	2	2			5	0.500			-1.25			4.00	1	0	1	0		1	1	1	5	0	1	0	1
1	1	0	0	0	0			5	-0.125	2	0.000	0.50	0.50		4.00	0	0	0	0	1	0	0	0	1	0	0	1	0
0	1		0	0	0			3	0.100	1	0.100	0.50	0.50		4.00	0	0	0	0	0	0	0	0	0	0	0	0	0
0	1		0	0	0			2	0.000						4.00	0	0	0	0	0	0	0	0	0	0	0	0	0
1	1	1	0	0	0		100.00	3	0.100	1	0.100	0.25	0.00		4.00	1	0	0	0	0	0	0	1	2	0	0	0	0
0	1	1	1	0	0		50.00	4	0.100			1.00	2.25		2.50	0	1	0	0		0	1	0	2	0	0	0	0
0	1	0	0	0	0		50.00	2	0.000						4.00	0	0	0	0		0	0	0	0	0	0	0	0
1	1		0													0	1	1	0	0	1	0	0	3	1	1	0	0
1	1		0	2	2		66.70	2	0.000			1.00	1.00		4.00	0	1	1	0		0	1	0	3	1	1	0	0
0	1	0	0	0	0			2	0.000	1	0.200	0.50	1.00	3	4.00	0	0	0	0	0	0	0	0	0	0	0	0	0
1	1	1	1	0	0		100.00	2	0.100					1	3.00	0	1	1	0	1	0	0	0	3	1	1	1	0

VFUMN	COMBDVNV	TotUNMET3	Totumncvi3	combifup	FUPCSUMN	FUPBVUMN	FUPODUMN2	FUPVFUMN	FUPCOMBDVNV	FUPTOTUMN3	fup3unmet	Initiatesengagement1	InitiatesEngagement2	Teacherspositivecomment1	TeachersPositiveComments2	Offtask1	OffTask2	Notvisuallyengaged1	NotVisuallyEngaged2	Repetitivebehaviours1	Repetitivebehaviours2
0	0	0	1	0	0	0	0	0	0	0	1	0	3	0	0	17	2	7	0	11	1
1	1	3	3	1	0	0	0	1	1	3	3	0	1	6	9	1	0	0	3	0	0
0	0	1	1	0	1	0	0	0	0	1	1	13	1	0	4	6	6	0	0	0	0
0	0	0	0	0	0	0	0	0	0	0	0	5	1	8	27	0	1	0	1	0	0
0	0	1	2	0	0	0	0	0	0	0	0										
0	0	0	0	0	0	0	0	0	0	0	0	4	3	11	5	2	0	0	0	0	0
0	0	0	0	0	0	0	0	0	0	0	0	1	1	14	11	2	0	0	0	0	0
0	1	1	2	0	0	0	0	0	0	0	0	2	2	3	7	0	0	0	0	0	0
0	0	1	1	0	0	0	0	0	0	0	0	0	1	16	22	3	1	0	0	0	0
0	1	3	3	1	0	0	0	0	0	1	1	4	3	12	5	1	0	0	0	0	0
0	0	2	2	1	0	0	0	0	0	1	1	2	3	1	16	1	6	0	3	20	4
0	0	0	0	0	0	0	0	0	0	0	0	0	2	0	9	2	4	2	1	4	1
0	1	1	1	0	0	0	0	0	1	1	1	0	1	0	11	8	0	0	0	0	0
0	0	0	0	0	0	0	0	0	0	0	0	0	1	4	4	9	2	0	0	0	0
0	0	1	1	0	0	0	0	0	0	0	0	0	3	2	10	7	9	3	9	16	8
0	0	0	0	0	0	0	0	0	0	0	0	2	1	1	10	2	0	0	0	9	9
0	1	1	1	0	0	0	0	0	1	1	1	1	5	0	4	6	0	0	0	0	0
0	0	1	1	1	0	0	0	0	0	1	1	3	2	20	14	3	0	0	4	0	1
0	0	0	0	0	0	0	0	0	0	0	0	0	0	13	2	2	12	0	1	5	0
0	0	1	1	0	0	0	0	0	0	0	0	1	1	13	4	2	4	6	0	0	6
0	0	0	0	0	0	0	0	0	0	0	0	0	5	12	4	1	0	0	0	0	0
0	0	2	2	0	0	0	0	0	0	0	0	0	2	4	5	0	0	0	0	0	0
0	0	0	0	0	0	0	0	0	0	0	0	10	5	1	0	0	0	0	0	0	0
0	0	0	0	0	0	0	0	0	0	0	0	0	4	22	16	0	2	5	0	10	0
0	0	0	0	0	0	0	0	0	0	0	0	6	7	0	1	0	1	0	0	0	0
0	0	0	1	0	0	0	0	0	0	0	0	6	13	2	3	3	0	0	0	1	0
0	0	2	2	0	0	0	0	0	0	0	0	3	2	21	2	2	0	0	0	1	0
0	0	0	0	0	0	0	0	0	0	0	0	2		13		3		0		1	
0	0	1	1	0	0	0	0	0	0	0	0	2	0	1	1	1	2	0	0	2	0
0	0	0	0	0	0	0	0	0	0	0	0	2	1	18	2	4	4	0	0	3	6
0	0	1	1	0	1	0	0	0	0	1	1	14	11	4	1	2	0	0	0	0	1
0	0	0	0	0	0	0	0	0	0	0	0	0	0	2	1	1	0	0	0	5	3
0	1	2	2	1	0	0	0	0	0	1	1	0	0	9	2	2	0	0	0	3	3
0	0	2	2	0	0	0	0	0	0	0	0	5	2	0	11	0	1	0	0	0	0
0	0	0	0	0	0	0	0	0	0	0	0	8	2	10	16	0	0	0	0	0	0
0	0	1	1	0	0	0	0	0	0	0	0	0	0	3	0	0	0	0	7	5	0
0	0	1	1	0	1	0	0	0	0	1	1	5	3	1	8	0	0	0	0	8	6
0	0	0	0	0	0	0	0	0	0	0	0	13	12	0	0	0	0	0	0	10	10
0	0	1	1	1	0	0	0	0	0	1	1	1	6	3	0	0	0	0	0	0	9
0	0	0	0	0	0	0	0	0	0	0	0	1	0	2	0	3	0	1	0	6	0
0	0	0	0	0	0	0	0	0	0	0	0	2	7	12	4	4	0	0	0	0	0
1	0	1	1	0	0	0	0	0	0	0	0	5	1	4	3	5	0	0	0	0	0
0	0	0	1	0	0	0	0	0	0	0	1	1	0	2	5	3	1	1	0	6	11
0	0	0	0	0	0	0	0	0	0	0	0	0	0	1	5	10	3	0	0	10	10
0	0	0	0	0	0	0	0	0	0	0	0	3	3	0	0	0	0	0	0	0	0
0	0	0	0	0	0	0	0	0	0	0	0	4	6	1	0	0	0	0	0	0	0
0	0	0	0	0	0	0	0	0	0	0	0										
0	0	0	0	0	0	0	0	0	0	0	0	1	3	1	0	0	0	0	0	0	0
0	0	0	0	0	0	0	0	0	0	0	0	2	3	1	3	0	0	0	0	0	0
0	0	3	3	1	1	0	0	0	0	2	2	1	2	13	10	0	0	0	0	0	0
0	0	0	0	0	0	0	0	0	0	0	0	0	2	2	0	9	0	0	0	12	0
0	1	2	2	1	0	0	0	0	0	1	1	0	0	5	0	0	0	0	0	0	0
0	0	0	0	0	0	0	0	0	0	0	0	5	3	14	1	0	0	0	0	4	0
0	0	1	1	1	0	0	0	0	0	1	1	0	2	0	0	2	0	0	0	3	0
0	0	0	0	0	0	0	0	0	0	0	0	11	2	2	0	0	1	0	0	0	1
0	0	0	0	0	0	0	0	0	0	0	0	0	1	0	0	1	0	0	0	0	0
0	0	0	1	0	0	0	0	0	0	0	0	1	0	0	0	0	5	0	0	8	8
0	0	0	0	0	0	0	0	0	0	0	0	0	2	0	0	4	0	0	0	0	1
0	0	1	2	0	0	0	0	0	0	0	1	6	4	2	0	0	0	0	0	0	0
0	0	0	0	0	0	0	0	0	0	0	0	1	1	0	1	0	0	0	0	0	0
0	0	0	0	0	0	0	0	0	0	0	0	5	3	1	4	0	0	0	0	0	0
0	0	0	0	0	0	0	0	0	0	0	0	6	9	0	0	5	0	2	0	0	5
0	0	0	0	0	0	0	0	0	0	0	0	7	3	2	1	0	0	0	0	0	0
0	0	0	0	0	0	0	0	0	0	0	0	2	0	0	0	0	0	0	0	0	1
0	0	2	2							0	0										
0	0	0	0	0	0	0	0	0	0	0	0	8	1	0	1	2	2	0	0	0	0
0	0	0	0	0	0	0	0	0	0	0	0	3	2	2	0	2	0	0	0	6	
0	0	0	1	0	0	0	0	0	0	0	1	6	10	1	2	0	0	0	0	0	0
0	0	0	0	0	0	0	0	0	0	0	0	10	4	6	0	0	0	0	0	0	0
0	0	0	0	0	0	0	0	0	0	0	0	0	1	0	0	0	0	0	0	0	0
0	0	0	0	0	0	0	0	0	0	0	0	2	5	2	0	1	0	0	0	0	0
0	0	0	1	0	0	0	0	0	0	0	1	4	1	1	4	1	0	0	0	0	0
0	0	0	0	0	0	0	0	0	0	0	0	0	0	0	0	1	1	0	0	6	0
0	0	0	0	0	0	0	0	0	0	0	0	12	3	0	0	0	0	0	0	0	0
0	0	0	0	0	0	0	0	0	0	0	0	5	1	0	2	0	0	0	0	0	0
0	0	0	0	0	0	0	0	0	0	0	0	2	2	0	1	0	0	0	0	0	0
0	0	0	0	0	0	0	0	0	0	0	0	5	2	0	1	3	0	0	1	0	0
0	0	0	0	0	0	0	0	0	0	0	0	7	3	0	1	0	2	0	0	0	0
0	0	0	0	0	0	0	0	0	0	0	0	5	2	3	1	0	3	0	0	0	0
0	0	0	0	0	0	0	0	0	0	0	0	3	7	3	0	1	0	0	0	1	0
0	0	1	1	0	0	0	0	0	0	0	0	5	7	3	4	0	0	0	0	0	0
0	0	0	0	0	0	0	0	0	0	0	0	4	2	3	0	0	0	0	0	0	0
0	0	0	0	0	0	0	0	0	0	0	0	1	2	0	0	4	0	0	0	0	0
0	0	0	0	0	0	0	0	0	0	0	0	9	6	1	0	0	0	0	0	0	0
0	0	0	0	0	0	0	0	0	0	0	0	10	12	1	0	0	2	0	0	0	0
0	0	0	0	0	0	0	0	0	0	0	0	8	8	3	6	0	0	0	0	0	0
0	0	0	1	0	0	0	0	0	0	0	1	5	3	1	0	0	3	0	0	0	0
0	0	0	1	0	0	0	0	0	0	0	0	0	3	3	4	0	0	0	0	0	0
0	0	1	1	0	0	0	0	0	0	0	0	3	5	0	0	2	0	0	0	0	0
0	0	0	0	0	0	0	0	0	0	0	0	9	3	1	1	0	0	0	0	0	0
0	0	0	0	0	0	0	0	0	0	0	0	3	5	0	0	0	0	0	0	0	0
0	0	0	0	0	0	0	0	0	0	0	0	11	3	3	0	1	3	0	0	0	0
0	0	0	0	0	0	0	0	0	0	0	0	6	8	2	1	1	0	0	0	0	0
0	0	0	0	0	0	0	0	0	0	0	0	5	2	0	0	0	1	0	0	0	0
0	0	2	2	0	0	0	0	0	0	0	0										
0	0	0	0	0	0	0	0	0	0	0	0	2	7	3	3	0	0	0	0	0	0
0	0	0	0	0	0	0	0	0	0	0	0	6	0	3	2	0	0	0	0	0	0
0	0	1	1	0	0	0	0	0	0	0	0	0	2	0	0	0	0	0	0	0	0
0	0	0	0	0	0	0	0	0	0	0	0	4	4	1	2	0	0	0	0	0	0
0	0	1	1	0	0	0	0	0	0	0	0	0	5	0	0	1	6	0	0	0	0
0	0	0	0	0	0	0	0	0	0	0	0	3	1	0	0	1	1	0	0	5	0

0	0	0	1	0	0	0	0	0	0	0	0	1	4	0	4	3	0	0	0	0	0
0	0	0	1	0	0	0	0	0	0	0	1	7	4	0	0	5	2	0	0	0	0
0	0	1	1	1	0	0	0	0	0	1	1	8	1	0	0	10	0	0	0	0	0
0	0	0	0	0	0	0	0	0	0	0	0	0	2	3	0	0	0	0	0	0	0
0	0	0	0	0	0	0	0	0	0	0	0	8	5	0	1	3	0	0	0	0	0
0	0	0	0	0	0	0	0	0	0	0	0	6	4	2	3	0	0	0	0	0	0
0	0	1	1	0	0	0	0	0	0	0	0	8	4	5	2	0	0	0	0	0	0
0	0	1	1	1	0	0	0	0	0	1	1	6	2	5	3	0	5	0	0	0	1
0	0	0	1	0	0	0	0	0	0	0	0	3	17	1	0	10	6	0	0	0	0
0	0	0	1	0	0	0	0	0	0	0	0	3	1	2	0	0	1	0	2	0	0
0	0	0	1	0	0	0	0	0	0	0	0	6		4		4				7	
0	0	0	1	0	0	0	0	0	0	0	0	7	3	1	1	3	0	0	0	0	0
0	0	0	0	0	0	0	0	0	0	0	0	2	4	0	0	3	0	0	0	0	10
0	0	3	3	0	0	0	0	0	0	0	0	5	1	1	0	2	7	0	0	2	0
0	0	0	1	0	0	0	0	0	0	0	0	0	9	2	2	0	0	0	0	0	0
0	0	0	1	0	0	0	0	0	0	0	0	1	0	2	0	1	0	0	0	2	0
0	0	0	0	0	0	0	0	0	0	0	0	3	9	1	0	1	1	0	0	0	0
0	0	0	0	0	0	0	0	0	0	0	0	3	7	5	2	0	1	0	0	0	5
0	0	0	0	0	0	0	0	0	0	0	0	3	3	3	1	0	2	0	0	0	0
0	0	1	1	1	0	0	0	0	0	1	1	1	5	2	2	0	0	0	3	2	0
1	0	3	4	0	0	0	0	0	0	0	0	3	1	0	0	0	3	0	1	2	0
0	0	2	2	0	0	0	0	0	0	0	0	2	4	0	2	4	0	2	0	0	0
0	0	0	1	0	0	0	0	0	0	0	0	5	11	1	1	1	0	0	0	0	0
0	0	1	2	0	0	0	0	0	0	0	0	3	1	4	1	1	3	0	0	0	0
0	0	0	1	0	0	0	0	0	0	0	0	2	3	1	1	0	0		0	0	0
0	0	0	0	0	0	0	0	0	0	0	0	9	13	4	0	0	3	0	0	0	0
0	0	0	0	0	0	0	0	0	0	0	0	0	1	3	0	6	5	0	0	4	10
0	0	0	1	0	0	0	0	0	0	0	0	3	3	1	1	0	0	0	0	3	0
0	0	0	1	0	0	0	0	0	0	0	1	8	10	0	0	1	0	0	0	0	0
0	0	0	0	0	0	0	0	0	0	0	0	3	5	0	0	2	6	0	0	0	1
0	0	0	1	0	0	0	0	0	0	0	1	2	6	2	0	0	4	0	0	0	0
0	0	0	0	0	0	0	0	0	0	0	0	1	4	4	4	0	0	0	0	0	0
0	0	0	0	0	0	0	0	0	0	0	0	1	3	8	1	3	3	0	0	3	1
0	0	0	0	0	0	0	0	0	0	0	0	5	9	2	0	0	0	0	0	0	0
0	0	0	0	0	0	0	0	0	0	0	0	0	2	3	0	2	3	0	0	0	1
0	0	1	1	0	0	1	0	0	0	1	1	3	9	3	1	2	0	0	0	0	0
0	0	0	1	0	0	0	0	0	0	0	1	6	2	3	1	4	0	0	0	0	0
0	0	0	0	0	0	0	0	0	0	0	0	6	5	3	2	0	5	0	0	0	0
0	0	0	0	0	0	0	0	0	0	0	0	3	1	0	0	3	1	0	0	0	0
0	0	0	0	0	0	0	0	0	0	0	0	5	2	2	6	0	0	0	0	0	0
0	0	1	1	0	0	0	0	0	0	0	0	5	3	1	0	5	0	0	0	0	0
0	0	2	3	1	0	0	0	0	0	1	1	1	7	3	2	4	2	0	0	0	0
0	0	1	2	0	0	0	0	0	0	0	0	5	8	0	0	4	2	3	1	0	0
0	0	0	1	0	0	0	0	0	0	0	0	4	0	3	1	2	0	0	1	2	0
0	0	0	0	0	0	0	0	0	0	0	0	7	3	1	2	8	0	0	0	0	0
0	0	0	0	0	0	0	0	0	0	0	0	3	2	1	1	0	0	0	0	0	0
0	0	0	0	0	0	0	0	0	0	0	0	6	2	1	3	1	0	1	2	0	0
0	0	0	0	0	0	0	0	0	0	0	0	8	1	0	0	0	0	0	0	0	0
0	0	1	1	0	0	0	0	0	0	0	0	1	9	0	0	0	0	0	0	0	0
0	0	0	0	0	0	0	0	0	0	0	0	9	9	0	2	0	0	0	0	0	0
0	0	1	1	0	0	0	0	0	0	0	0	4	1	2	0	2	0	1	3	9	5
0	0	0	0	0	0	0	0	0	0	0	0	3	1	2	0	0	0	0	0	0	0
0	0	0	0	0	0	0	0	0	0	0	0	9	2	10	3	0	0	1	0	0	0
0	0	0	0	0	0	0	0	0	0	0	0	4	2	7	0	0	0	0	0	0	6
0	0	0	0	0	0	0	0	0	0	0	0	0	1	32	7	1	0	0	0	8	9
0	0	0	0	0	0	0	0	0	0	0	0	3	3	6	7	0	0	0	0	0	3
0	0	1	2	0	0	0	0	0	0	0	0	1	0	5	9	2	2	0	1	0	4
0	0	0	1	0	0	0	0	0	0	0	0	10	4	0	5	0	0	0	1	0	1
0	0	0	1	0	0	0	0	0	0	0	0	2	2	2	9	1	0	0	0	9	10
0	0	1	1	0	0	0	0	0	0	0	0	2	1	2	0	0	1	0	4	0	0
0	0	0	1	0	0	0	0	0	0	0	0	4	2	7	4	0	1	0	0	1	0
0	0	0	1	0	0	0	0	0	0	0	0	1	1	5	1	0	0	0	0	0	0
0	0	1	2	0	0	0	0	0	0	0	0	2	1	5	0	0	0	0	0	0	0
0	0	2	3	0	0	0	0	0	0	0	0	6	0	3	8	0	2	0	0	0	0
0	0	0	0	0	0	0	0	0	0	0	0	0	0	0	4	3	0	0	0	0	0
0	0	0	0	0	0	0	0	0	0	0	0	0	0	7	6	4	4	0	0	0	3
0	0	0	0	0	0	0	0	0	0	0	0	2	5	1	0	1	0	2	0	0	3
0	0	1	1	0	0	0	0	0	0	0	0	6	3	4	2	0	1	0	0	0	0
0	0	0	0	0	0	0	0	0	0	0	0	3	4	5	1	0	0	0	0	0	0
0	0	0	1	0	0	0	0	0	0	0	1	7	1	1	0	0	3	0	0	0	0
0	0	0	0	0	0	0	0	0	0	0	0	3	1	0	0	0	1	0	3	0	2
0	0	0	0	0	0	0	0	0	0	0	0	11	5	5	0	0	0	0	0	1	0
0	0	0	0	0	0	0	0	0	0	0	0	7	10	18	12	0	0	0	0	0	0
0	0	0	0	0	0	0	0	0	0	0	0	1	0	15	13	1	5	0	1	8	7
0	0	2	2	1	1	0	0	0	0	2	2	0	2	5	3	0	1	0	0	7	9
0	0	0	0	0	0	0	0	0	0	0	0										
0	0	0	0	0	0	0	0	0	0	0	0	0	14	8	1	0	8	0	0	1	
0	0	1	1	0	1	0	0	0	0	1	1	6	2	21	22	1	0	0	0	0	0
0	0	0	0	0	0	0	0	0	0	0	0	4	1	1	1	0	0	0	1	0	0
1	0	2	2	1	0	0	0	1	0	2	2	0	0	6	3	0	2	3	0	0	0
0	0	0	1	0	0	0	0	0	0	0	0	3	2	0	1	0	1	0	0	0	0
0	0	0	0	0	0	0	0	0	0	0	0	9	3	3	3	0	0	0	0	0	0
0	0	0	0	0	0	0	0	0	0	0	0	2	1	10	1	2	1	0	0	5	4
0	0	0	0	0	0	0	0	0	0	0	0	4	0	3	8	1	0	0	0	7	7
0	1	3	4	1	1	0	0	0	1	3	4	1	1	11	20	1	0	0	0	0	0
0	0	0	0	0	0	0	0	0	0	0	0	1	0	5	16	3	0	0	0	0	2
0	0	0	0	0	0	0	0	0	0	0	0	0	0	8	9	0	0	0	0	0	0
0	0	0	0	0	0	0	0	0	0	0	0	4	8	3	12	0	3	0	0	0	4
0	1	3	4	0	0	0	0	0	0	0	0	1	10	1	1	0	0	0	0	0	1
0	0	1	1	0	0	0	0	0	0	0	0	2	3	9	17	0	0	0	0	0	0
0	0	0	0	0	0	0	0	0	0	0	0	1	9	4	0	0	4	0	2	0	0
0	0	0	0	0	0	0	0	0	0	0	0	7	0	7	4	0	3	0	2	0	0
0	1	1	2	0	0	0	0	0	0	0	0	5	0	1	4	0	1	0	0	0	0
0	0	0	0	0	0	0	0	0	0	0	0	2	1	1	0	0	0	0	0	0	0
0	0	0	0	0	0	0	0	0	0	0	0	3	1	0	3	0	4	0	0	6	0
0	0	2	2	1	0	0	0	0	0	1	1	0	2	6	3	2	3	0	0	0	0
0	0	2	2	0	0	0	0	0	0	0	0	6	2	6	3	1	0	0	0	0	0
0	0	0	0	0	0	0	0	0	0	0	0	6	5	1	2	0	0	0	0	0	1
0	0	3	3	0	0	0	0	0	0	0	0	0	3	5	4	5	2	4	0	0	0
